# Phytochemical Composition, Cytoprotective and Antioxidant Activity of *Werneria glaberrima* Against H_2_O_2_-Induced Oxidative Stress in Human SH-SY5Y Neuroblastoma Cells

**DOI:** 10.3390/molecules31203765

**Published:** 2026-10-20

**Authors:** Duxan Arancibia, Matías Lira, Bastian Alcota, Mario J. Simirgiotis, Adrián Paredes, Rafaella V. Zárate

**Affiliations:** 1 Departamento de Ciencias Farmacéuticas, Facultad de Ciencias, Universidad Católica del Norte, Antofagasta 1270709, Chile; duxan.arancibia@ucn.cl (D.A.); bastianalcotagonzalez@gmail.com (B.A.); 2 Instituto de Ciencias de la Salud, Universidad de O’Higgins, Rancagua 2841959, Chile; matias.lira@uoh.cl; 3 Instituto de Farmacia, Facultad de Ciencias, Universidad Austral de Chile, Valdivia 5110566, Chile; mario.simirgiotis@uach.cl; 4 Instituto Antofagasta (IA), Universidad de Antofagasta, Antofagasta 1270300, Chile; 5 Departamento Biomédico, Facultad de Ciencias de la Salud, Universidad de Antofagasta, Antofagasta 1270300, Chile

**Keywords:** *Werneria glaberrima*, oxidative stress, *ent*-kaurenoic acid, acetophenone, benzofuran, SH-SY5Y, medicinal plants

## Abstract

Plants from extreme environments such as the Atacama Desert are an underexplored source of novel bioactive metabolites, since intense UV radiation, thermal amplitude, and water scarcity force resident flora to rely on specialized biochemical defenses. *Werneria glaberrima*, a high-Andean species used in northern Chile as a traditional medicinal plant for uterine pain and to facilitate childbirth, is chemically characterized by prenylated acetophenones, benzofuran derivatives, and triterpenes. Although antioxidant activity has been shown in the taxonomically related high-Andean species *Senecio nutans*, the antioxidant and redox-modulating activity of *W. glaberrima* remains unexplored. Oxidative stress, an imbalance between reactive oxygen species (ROS) production and antioxidant capacity, contributes to the pathophysiology of cancer, cardiovascular disease, diabetes, and neurodegenerative disorders. Here, we characterized the phytochemical composition of the total extract (TE) of *W. glaberrima* and evaluated the biocompatibility and cytoprotection of the TE and three isolated metabolites, Wg-1 (*ent*-kaurenoic acid), Wg-2 (dihydrobenzofuran 6-hydroxytremetone), and Wg-3 (4-hydroxy-3-prenylacetophenone), in human SH-SY5Y neuroblastoma cells. We also assessed their ability to attenuate the intracellular pro-oxidant environment induced by hydrogen peroxide (H_2_O_2_). The TE was biocompatible and cytoprotective, whereas Wg-2 showed the broadest biocompatibility window among the isolated metabolites. Pretreatment with TE or Wg-1 protected SH-SY5Y cells against damage induced by different H_2_O_2_ concentrations, and decreased H_2_O_2_-induced DCF oxidation at the concentration tested. These results provide the first chemical characterization of the TE of *W. glaberrima* and initial in vitro evidence of the cytoprotective and antioxidant potential of this extract and Wg-1.

## 1. Introduction

Natural products (NPs) have been shown to modulate multiple intracellular signaling pathways in mammals, bridging plant ecology with human health [1]. In this regard, various plant-derived secondary metabolites, including terpenoids, phenolic acids, alkaloids, saponins, and glycosides, have demonstrated a broad spectrum of biological activities, encompassing antimalarial, anti-inflammatory, antioxidant, analgesic, anticancer, and antimicrobial effects [2].

Extreme environments impose a distinctive selective pressure on the species that manage to survive under those conditions, fostering a unique chemodiversity. The Atacama Desert, the driest non-polar desert on Earth, combining intense UV radiation, wide thermal amplitude, hypoxia, and water scarcity across an altitudinal gradient from roughly 2500 to 4500 m a.s.l., forces resident flora to rely on specialized biochemical defenses for survival, yielding a metabolome enriched in stress-protective compounds not typically found in lowland taxa [3,4]. This makes high-Andean Atacama plants a comparatively under-exploited reservoir for bioprospecting and a rational starting point when searching for novel chemotypes of bioactive metabolites.

The genus *Werneria* (Asteraceae, tribe Senecioneae, subtribe Senecioninae) was first described in 1818 by Carl S. Kunth and reviewed in [5]. The morphological variability observed across species within this genus undermines its status as a monophyletic group, and on this basis nearly half of its species have since been reassigned to the newly established genus *Xenophyllum* [6]. As currently circumscribed, genus *Werneria* likely comprises 25–30 species [7], distributed throughout the South American Andes above 2800 m a.s.l., ranging from Venezuela, Colombia, Ecuador, Peru, and Bolivia to northern Argentina and Chile [5]. The *Werneria* genus has traditionally been used for altitude mountain sickness, hypertension, and gastrointestinal disorders [8]. *Werneria nubigena* is used as a tea because of its digestive and antihelmintic properties [9]. The whole plant decoction of *Werneria villosa* is traditionally used for improving blood circulation and to treat general body pain and uterine swelling [10]. Other traditional uses include the treatment of digestive disorders, coughs, and bronchitis [11]. Furthermore, Martinez and colleagues reported that the *Werneria* genus is used in traditional Chilean medicine for the treatment of hypertension and “soroche” (mountain sickness) [12].

Phytochemical studies of several *Werneria* species have identified prenylated *p*-hydroxyacetophenones, benzofurans, chromenes, coumarates, and *ent*-kaurane and *ent*-labdane diterpenes. Some of these compounds have shown antimicrobial, anti-HIV, hypotensive, and photoprotective activities [13,14]. Despite these activities described for *Werneria* species, their antioxidant potential remains comparatively unexplored. The phenolic hydroxyl groups and conjugated aromatic systems present in some of these metabolites provide a structural basis for hypothesizing radical-scavenging or redox-modulating properties.

**Figure 1:**
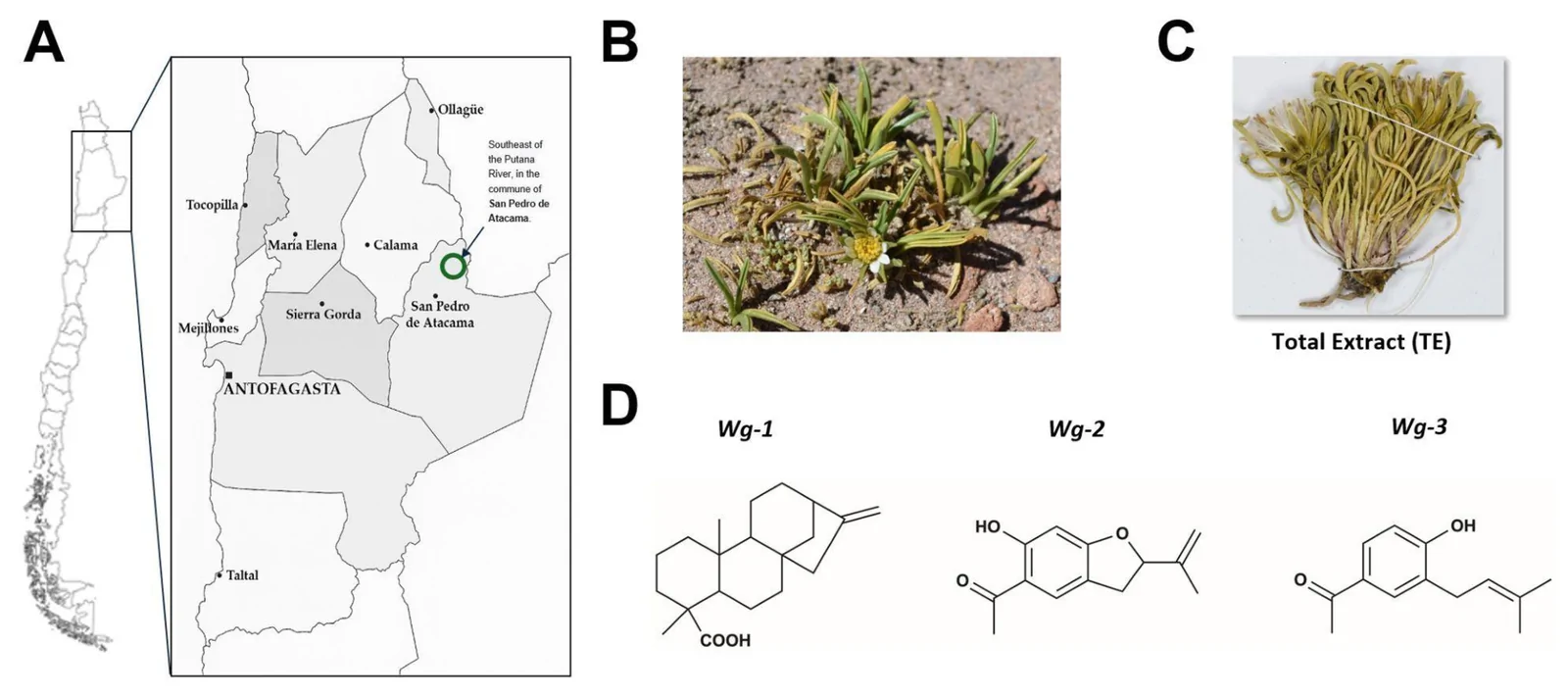
(**A**) Geographic distribution of *Werneria glaberrima* in northern Chile. The green circle indicates the specimen collection site; coordinates are provided in the Materials and Methods section (**B**) displays a picture of *W. glaberrima* in its native environment, and (**C**) the aerial parts used for the preparation of the Total Extract (TE). Obtained from Herbario Digital on https://www.herbariodigital.cl. Accessed on 6 October 2026. (**D**) The chemical structures of the three metabolites isolated.

*Werneria glaberrima* Phil. (local names “Maransel” and “Warillach’u”) grows between 4200 and 4400 m a.s.l. in the Antofagasta Region of northern Chile (Figure 1A), where it is used in traditional medicine for uterine pain, to facilitate childbirth, and for bladder conditions [5]. Its antioxidant activity and redox-modulating properties have not yet been explored. In this context, the taxonomically related high-Andean species *Senecio nutans* provides a relevant comparative precedent, as its extracts have shown in vitro antioxidant and antibacterial activity [15].

Antioxidant activity encompasses different mechanisms through which a compound or extract may limit oxidative damage. These include the direct scavenging of reactive oxygen species (ROS) by electron or hydrogen-atom transfer, the chelation of pro-oxidant metals, and the modulation of endogenous antioxidant defenses, including activation of the Nrf2 pathway [16]. This capacity is physiologically relevant because ROS are not merely damaging byproducts. At controlled concentrations, they act as signaling intermediates that regulate proliferation, differentiation, and immune responses [17,18,19]. However, their accumulation beyond the buffering capacity of endogenous antioxidant systems produces oxidative stress, leading to protein oxidation, lipid peroxidation, and DNA damage that may culminate in cell death [20]. Oxidative stress has been implicated in the pathophysiology of cancer [21], cardiovascular diseases [22], diabetes [23], and neurodegenerative diseases [24], underscoring the relevance of identifying antioxidant compounds and supporting the investigation of plant-derived metabolites capable of modulating cellular redox homeostasis.

Although some constituents of the genus *Werneria* have been previously identified, the phytochemical profile of root and aerial part extracts of *W. glaberrima* remains poorly characterized. Moreover, it is unknown whether the metabolites present in these extracts can modulate intracellular ROS levels or protect cells against oxidative damage. Therefore, to address these knowledge gaps, we characterized the phytochemical composition of *W. glaberrima* by UHPLC-ESI-QOrbitrap-MS and assessed the in vitro antioxidant activity of its total extract (TE) using standardized chemical assays. Moreover, in SH-SY5Y cells, we evaluated the cytoprotective effects and the ability to attenuate the H_2_O_2_-induced intracellular pro-oxidant environment of TE and three selected metabolites: *ent*-kaurenoic acid (Wg-1), 1-(2,3-dihydro-6-hydroxy-2-(1-methylethenyl)-5-benzofuranyl)ethenone (Wg-2), and 4-hydroxy-3-(3-methyl-2-butenyl)acetophenone (Wg-3). Their ability to attenuate the intracellular pro-oxidant environment induced by H_2_O_2_ in SH-SY5Y cells was assessed using DCFH-DA-derived fluorescence. This study expands the phytochemical knowledge of *W. glaberrima* and provides initial evidence regarding the antioxidant activity and cytoprotective potential of its extract and selected metabolites.

## 2. Results and Discussion

### 2.1. Phytochemical Study of Werneria glaberrima by UHPLC-ESI-QOrbitrap-MS

Phytochemical study of *Werneria* spp. has been reported to identify terpenoids as the main group of biologically active compounds. Among them, different subclasses have been described, including diterpenes and sesquiterpenes, together with flavonoids, benzofuran derivatives, and coumarins [8,14,25]. However, to the best of our knowledge, the phytochemical composition of *W. glaberrima* has not previously been characterized (Figure 1B,C). Therefore, a hydroalcoholic extract of *W. glaberrima*, hereafter referred to as total extract (TE), was prepared according to the methods described in Section 3.3.

**Figure 2:**
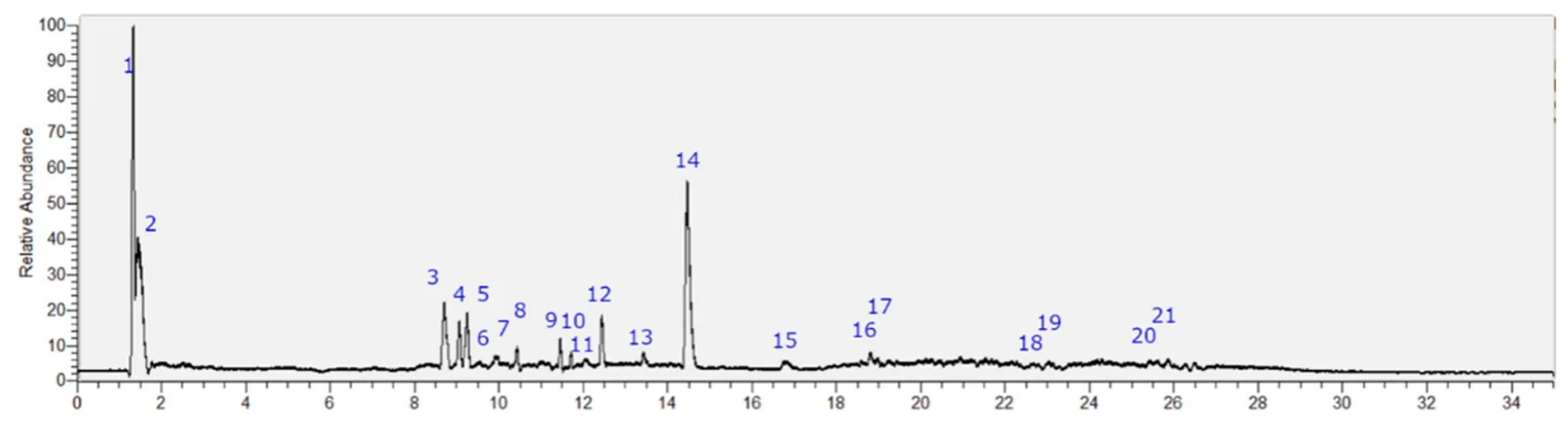
Total ion chromatogram (TIC) of the TE of *W. glaberrima* obtained by UHPLC-ESI-Q-Orbitrap-MS.

The phytochemical profile of TE was analyzed by UHPLC-ESI-QOrbitrap-MS (Figure 2). Chromatographic features were tentatively annotated based on retention time, accurate mass measurements, proposed molecular formulas, and MS/MS fragmentation patterns, together with comparisons with spectral databases and previously reported data in the literature. A total of 21 compounds were tentatively annotated. In terms of chemical diversity, phenolic acids constituted the most represented class, with seven compounds assigned, followed by fatty acids, with four compounds. Flavonoids constituted the third most represented group, including three flavonols: kaempferol, kaempferol-3-O-rutinoside, and combretol. Additional chemical classes included the presence of diterpenes, sugar/glycoside derivatives, a stilbene glycoside (rhapontin), and an iridoid glucoside (asperuloside). A naphthylacetic acid derivative was also tentatively annotated. Detailed information on all detected compounds is presented in Table 1, while their distribution across chemical classes, based on the number of tentatively annotated compounds, is summarized in Supplementary Information: Figure S1.

The chromatographic assignments are discussed according to metabolite class.

#### 2.1.1. Phenolic Acids

Peak 1 with a pseudomolecular ion at *m*/*z*: 191.05646 was identified as quinic acid; peak 5 with a pseudomolecular ion at *m*/*z*: 353.08923 as chlorogenic acid (C_16_H_17_O_9_) and a daughter quinic acid ion at *m*/*z*: 191.05631; peak 8 as neochlorogenic acid; peak 9 as its dicaffeoyl derivative cynarine (C_25_H_24_O_12_); peak 11 as salicortin (C_27_H_29_O_13_); peak 14 as 3,4-dimethoxycinnamic acid (C_11_H_11_O_4_); and finally, peak 15 was assigned as acetylsalicylsalicylic acid (C_16_H_11_O_6_). Peak 19 (C_30_H_37_O_4_) was tentatively assigned to Kassinol K, a natural polyphenol isolated from plants (such as the genus *Broussonetia* or mulberry trees) and studied for their antioxidant and bioactive properties [25].

#### 2.1.2. Terpenes

Peak 7, with a pseudomolecular ion at *m*/*z*: 413.10770, was identified tentatively as asperuloside (C_18_H_21_O_11_), an iridoid glucoside widely distributed in the Rubiaceae and Verbenaceae families but rarely documented within Asteraceae. To our knowledge, this represents the first tentative report of asperuloside in *W. glaberrima*. Peak 20 was isolated and identified as *ent*-kaur-16-en-19-oic acid (C_20_H_29_O_2_), and peak 21 as C_20_H_29_O_3_ is an unknown terpene.

#### 2.1.3. Fatty Acids

Peak 3 with an ion at *m*/*z*: 293.12558 was identified as 4,7,10,13-tetraoxahexadecanedioic acid (C_12_H_21_O_8_), peak 16 as tetracosanoic acid (C_24_H_47_O_2_) and peak 18 as arachidic acid (C_20_H_39_O_2_).

#### 2.1.4. Sugars

Peak 4 with a pseudomolecular ion at 425.16828 was identified as foliachinenoside H (C_17_H_29_O_12_), and peak 17 as atractyloside-A-14-O-β-D-fructofuranoside.

#### 2.1.5. Flavones

Peak 6 with a pseudomolecular ion at *m*/*z*: 593.15338 was assigned as kaempferol-3-O-rutinoside (C_27_H_29_O_15_), while peak 12 with a parent ion at 387.11005 as the polymethylated flavonol combretol (C_20_H_19_O_8_) and peak 13 as kaempferol (C_15_H_9_O_6_).

#### 2.1.6. Stilbenes

Peak 2, with a pseudomolecular ion *m*/*z*: 419.1347, was identified tentatively as rhapontin (C_21_H_23_O_9_), a resveratrol-derived stilbene glucoside. This class of secondary metabolite has not been previously reported for the genus *Werneria*.

#### 2.1.7. Naphthylacetic Acid Derivatives

Peak 10, with a pseudomolecular ion at *m*/*z*: 215.07224, was tentatively identified as 6-methoxy-2-naphthylacetic acid (C_13_H_11_O_3_). Naphthalene-type skeletons are uncommon among plant secondary metabolites, and this structure closely resembles synthetic arylacetic-acid scaffolds (e.g., naproxen-related compounds).

The metabolite profile obtained for *W. glaberrima* is broadly consistent with previous phytochemical surveys of the genus *Werneria*, which have repeatedly reported diterpenes (kaurane-type acids and related terpenoids) and *p*-hydroxyacetophenone and benzofuran derivatives as recurrent chemical markers [13,26], alongside pyrrolizidine alkaloids reported in *W. nubigena* [27]. The tentative annotation of *ent*-kaur-16-en-19-oic and a second possible unidentified diterpene (peak 21) in the TE of *W. glaberrima* supports the potential chemotaxonomic relevance of *ent*-kaurane-type diterpenoids for this genus. Also, the three flavonol derivatives annotated, kaempferol, kaempferol-3-O-rutinoside, and combretol, extend the flavonoid pattern already annotated for other *Werneria* species. However, the comparatively high representation of phenolic acids (mono- and dicaffeoylquinic acids, salicortin, dimethoxycinnamic, and acetylsalicylsalicylic acids) and fatty acids (medium- and long-chain saturated and oxygenated acids) among the tentatively annotated metabolites distinguishes the phytochemical profile of *W. glaberrima* from those previously described for other *Werneria* species.

Among the tentatively annotated compounds, two annotations deserve particular attention. Peak 19 was tentatively assigned as Kazinol K, a chromone previously described from plants of the genus *Broussonetia*, specifically the species *Broussonetia papyrifera* and *Broussonetia kazinoki* [25]. Similarly, peak 10 was tentatively assigned as 6-methoxy-2-naphthylacetic acid, which has a naphthalene skeleton that is uncommon among plants and is otherwise documented as a drug metabolite rather than as a naturally occurring phytochemical [28]. Although this compound has previously been reported in ethanolic leaf extracts of *Moringa oleifera* [29], its occurrence in plants remains to be established. Also, the co-occurrence of a stilbene glucoside (rhapontin), whose aglycone rhapontigenin has been reviewed for its metabolism and bioactivity [30], and an iridoid glucoside (asperuloside), a compound otherwise well characterized in Rubiaceae [31], were tentatively annotated for the first time in the *Werneria* genus. If confirmed, these findings could expand the known chemical diversity of the genus. However, their identities should be confirmed through targeted isolation and NMR analysis.

Also, three of interest were successfully isolated from *W. glaberrima* (Figure 1D): *ent*-kaurenoic acid (Wg-1), 1-(2,3-dihydro-6-hydroxy-2-(1-methylethenyl) -5-benzofuranyl)ethenone (Wg-2), and 4-hydroxy-3-(3-methyl-2-butenyl)acetophenone (Wg-3). Biocompatibility and potential antioxidant activity were evaluated in the TE and the isolated metabolites of *W. glaberrima*. The structure of these molecules has been confirmed by 1D/2D NMR (^1^H, ^13^C, HSQC/HMQC and HMBC) and FT-IR spectroscopy (Supplementary Information: Figures S2–S16).

### 2.2. In Vitro Characterization of Antioxidant Activity of Werneria glaberrima

The hydroalcoholic extract of *W. glaberrima* exhibited a total phenolic content of 234.6 ± 11.4 mg gallic acid equivalents (GAE)/g of dry extract and a total flavonoid content of 115.6 ± 8.76 mg quercetin equivalents (QE)/g of dry extract (Table 2). The ferric reducing antioxidant power (FRAP) was 192.7 ± 15.6 mg Trolox equivalents (TE)/g of dry extract. In the DPPH radical scavenging assay, the extract showed an IC_50_ of 284.4 ± 14.5 µg/mL and, against the ABTS•+ radical cation, an IC_50_ of 342.4 ± 12.4 µg/mL; these values were considerably higher (i.e., indicating lower potency) than those of the Trolox standard (IC_50_ DPPH = 8.75 ± 1.1 µg/mL; IC_50_ ABTS = 11.7 ± 1.8 µg/mL). The metal-chelating activity of the extract (IC_50_ = 116.7 ± 1.6 µg/mL) was also considerably lower than that of the EDTA standard (IC_50_ = 5.2 ± 0.79 µg/mL).

The phenolic and flavonoid content of the *W. glaberrima* TE, along with its reducing capacity (FRAP), indicates a matrix with a significant polyphenol load; however, its free radical scavenging activity (DPPH, ABTS) and metal-chelating capacity were considerably lower than those of the reference standards (Trolox and EDTA, respectively), suggesting moderate rather than outstanding antioxidant activity. To the best of our knowledge, there are no prior reports of in vitro antioxidant activity for *W. glaberrima*; thus, these results represent the first characterization of this property for the species. The available data regarding antioxidant activity within the genus pertains to *Xenophyllum poposum* (Phil.). Its methanolic extract showed an IC_50_ of 160 ppm against DPPH, but no data was reported regarding phenols, flavonoids, FRAP, or chelating activity [32].

At the family level, the accumulation of phenolic compounds and flavonoids in high-Andean Asteraceae species has been proposed as an adaptive response to the increased UV-B radiation characteristic of high altitudes. This radiation is thought to induce the synthesis of secondary metabolites that serve a photoprotective function and, secondarily, an antioxidant [33]. However, that same study shows that the magnitude of this response is species-specific rather than a universal pattern; therefore, this hypothesis should be presented as a possible ecological explanation for the observed phenolic profile, not as an established fact for *W. glaberrima*.

### 2.3. Biocompatibility of the TE and Selected Metabolites of W. glaberrima in SH-SY5Y Cells

**Figure 3:**
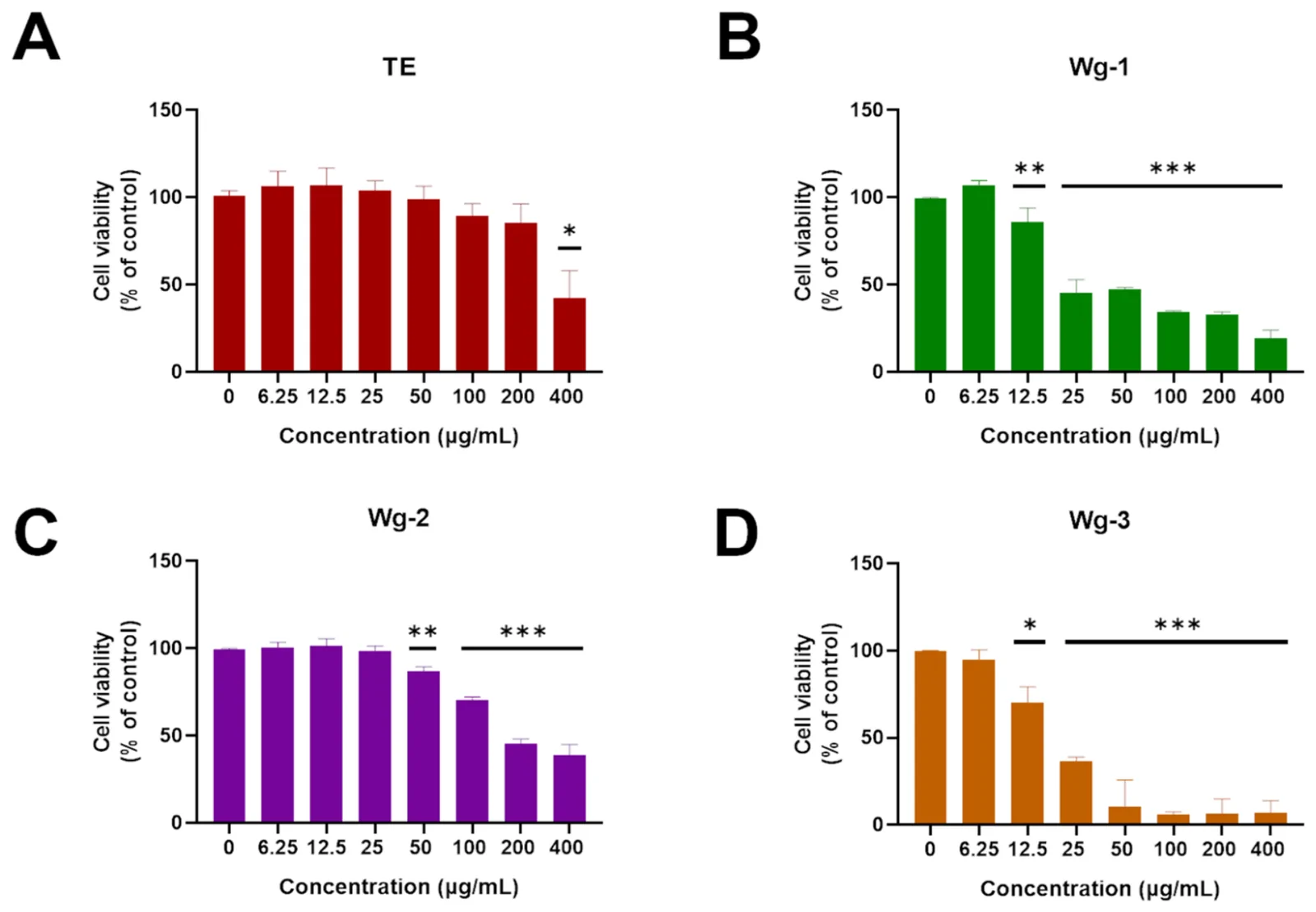
Cell viability of SH-SY5Y cells exposed to increasing concentrations of (**A**) total extract, (**B**) Wg-1, (**C**) Wg-2, and (**D**) Wg-3 for 24 h. The *Y*-axis shows the cell viability (% of the control). Concentration of 0 µg/mL corresponds to vehicle control. Data represents the mean ± SEM from three independent biological replicates (*n* = 3). Statistical significance of the differences compared with the values of the respective control group is indicated on each graph by * *p* < 0.05, ** *p* < 0.01, and *** *p* < 0.001.

The biocompatibility assessment on SH-SY5Y cells revealed concentration-dependent viability profiles that defined the concentration windows for subsequent cytoprotection experiments. The TE did not exhibit reduced cell viability at concentrations ranging from 6.25 to 200 µg/mL, whereas a significant cytotoxic effect was observed at 400 µg/mL (Figure 3A). This broad safety margin is particularly noteworthy given the chemical complexity of the TE, which contains phenolic acids, flavonoids, diterpenes, and fatty acids (Table 1). The sharp loss of viability at 400 µg/mL aligns with the well-documented hormetic behavior of plant polyphenolic mixtures, where high concentrations trigger pro-oxidant effects through metal-catalyzed autooxidation and subsequent H_2_O_2_ generation [34,35]. This phenomenon has been extensively characterized by green tea catechins, resveratrol, and quercetin, which exhibit biphasic dose–response curves in various cell models [35].

Among the isolated metabolites, Wg-1 (*ent*-kaurenoic acid) demonstrated one of the narrowest biocompatibility ranges, as it did not significantly reduce cell viability at 6.25 μg/mL, whereas a modest but significant reduction was observed at 12.5 μg/mL (Figure 3B). This finding aligns with previous reports in HEK293 and RAW264.7 macrophages, where *ent*-kaurenoic acid showed negligible cytotoxicity below 10 μM [36]. The *ent*-kaurane skeleton, characterized by its tetracyclic diterpenoid structure, appears to confer moderate biological activity without compromising membrane integrity at low micromolar concentrations [37].

Wg-2, a benzofuran derivative, displayed the widest biocompatibility window among the isolated compounds. It did not significantly reduce cell viability at concentrations up to 25 μg/mL, whereas a gradual concentration-dependent decline was observed at 50 μg/mL and above (Figure 3C). This pattern is consistent with cytotoxic effects reported for a synthetic benzofuran-linked chalcone in colon cancer cells through caspase-3-mediated apoptosis [38]. Although SH-SY5Y cells are derived from human neuroblastoma, and that compound is structurally distinct from Wg-2, the mechanism underlying the loss of cell viability observed at higher Wg-2 concentrations remains unknown. Importantly, the absence of cytotoxicity at 25 μg/mL allowed us to test Wg-2 at relevant concentrations without confounding cell death.

Wg-3, a prenylated acetophenone, exhibited a restricted biocompatibility window, with cell viability declining significantly from 12.5 μg/mL and above (Figure 3D). This profile mirrors the behavior of apocynin (4-hydroxy-3-methoxyacetophenone), which shares the *p*-hydroxyacetophenone scaffold with Wg-3 and has been reported to exert context-dependent redox effects. Although apocynin is commonly used to inhibit NADPH oxidase-dependent ROS production, it has also been shown to increase H_2_O_2_ levels, disrupt glutathione homeostasis, promote lipid peroxidation, and induce lactate dehydrogenase (LDH) release in certain cellular models [39]. Its oxidation potential and peroxidase-dependent reactivity further support the capacity of apocynin to participate in complex redox reactions [40]. However, because Wg-3 exhibits a prenyl substituent at C-3 instead of a methoxy group in apocynin, their physicochemical and biological properties may differ substantially. Prenylation may increase lipophilicity and interactions with cellular membranes, potentially facilitating membrane partitioning and the accumulation in intracellular compartments. However, the mechanism of the cytotoxicity of Wg-3 needs to be explored.

### 2.4. Cytoprotective Effects of TE and Selected Metabolites Against H_2_O_2_-Induced Cellular Damage

**Figure 4:**
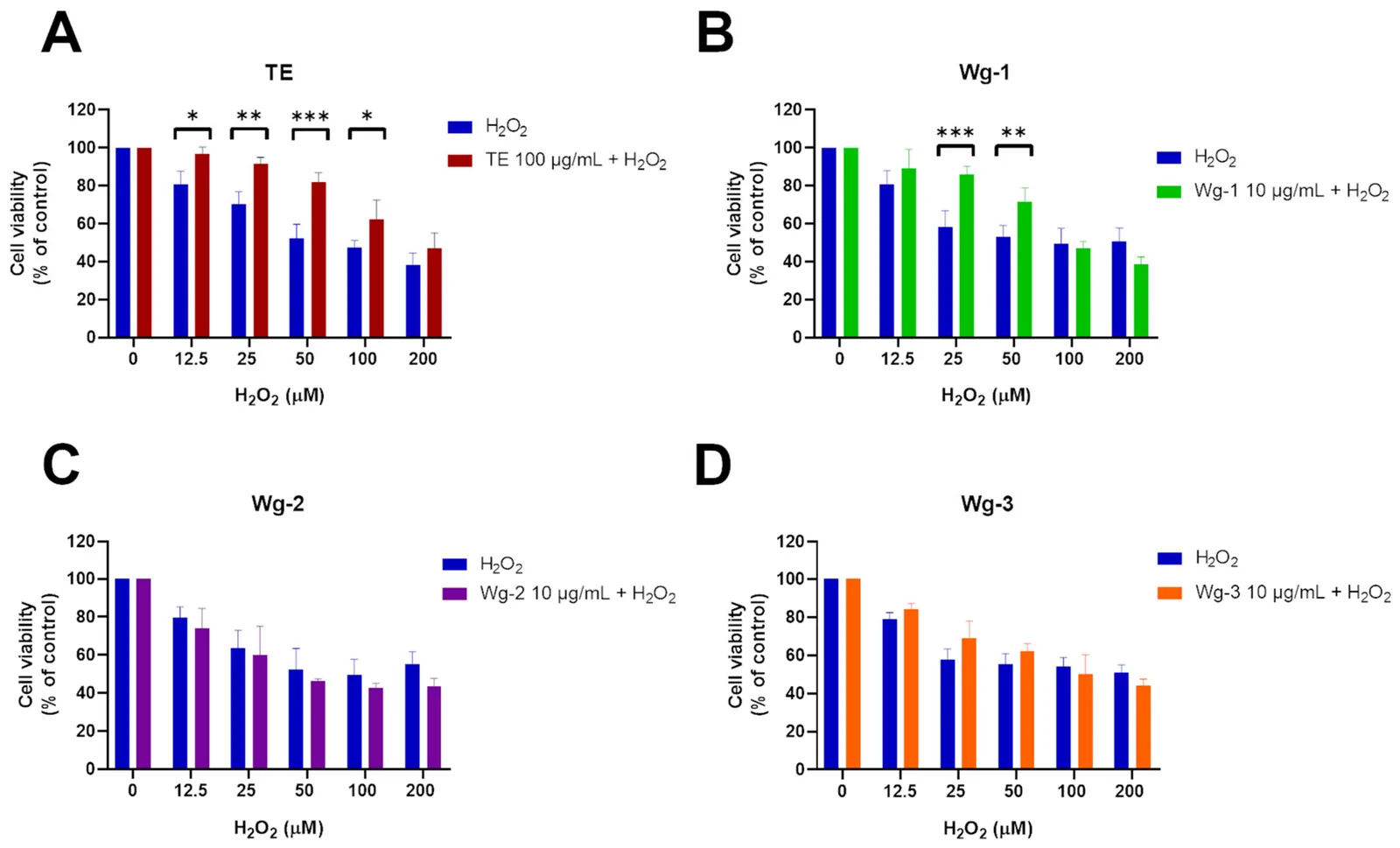
Cytoprotection of SH-SY5Y cells pretreated with a fixed concentration of (**A**) TE (100 µg/mL), (**B**) Wg-1, (**C**) Wg-2, and (**D**) Wg-3 (10 µg/mL) for 24 h, then exposed to increasing H_2_O_2_ (12.5–200 µM) for 2 h, compared with H_2_O_2_ alone. The *Y*-axis shows the cell viability (% of the control). Concentration of 0 µg/mL corresponds to vehicle control. Data represents the mean ± SEM from three independent biological replicates (*n* = 3). Statistical significance was determined by two-way ANOVA. Differences comparing the values with the respective control group are indicated on each graph by * *p* < 0.05, ** *p* < 0.01, *** *p* < 0.001.

The cytoprotection experiments employed a fixed-pretreatment design of the TE and metabolites Wg-1, Wg-2, or Wg-3 against increasing H_2_O_2_ concentrations to assess the robustness of protection. Pretreatment with TE (100 μg/mL) significantly preserved viability against 25, 50, and 100 μM H_2_O_2_, relative to the control without TE (Figure 4A) (H_2_O_2_ main F = 79.77; TE main F = 58.81; Interaction F = 4.43, *p* = 0.0053). However, protection declined at 200 μM H_2_O_2_. One possible explanation is that, at this concentration (200 μM), the oxidative challenge exceeded the protective capacity, i.e., a saturation of the extract’s neutralizing capacity, potentially due to a finite pool of antioxidant constituents. However, additional mechanistic studies are required to test this hypothesis. Importantly, the TE’s protective effect cannot be attributed solely to direct radical scavenging, as the extract’s chemical composition includes methylated polyphenols and complex glycosides that are poor “chemical” antioxidants but can still protect cells through indirect mechanisms [41]. This distinction is critical because many plant extracts may exert cytoprotection via Nrf2-mediated upregulation of endogenous antioxidant enzymes (e.g., HO-1, NQO1, GST) rather than through stoichiometric neutralization of H_2_O_2_ [42]. The tentative annotation of kaempferol glycosides and chlorogenic acid derivatives in the TE (Table 1) supports this possibility. Chlorogenic acids have been associated with Nrf2-dependent cellular responses, and specific kaempferol derivatives have been shown to protect SH-SY5Y cells against H_2_O_2_-induced injury while increasing Nrf2 expression [43,44]. The 24 h pretreatment period employed in our study might allow sufficient time for transcriptional reprogramming; however, this mechanistic hypothesis requires assessment.

Among the isolated metabolites, pretreatment with Wg-1 (*ent*-kaurenoic acid; 10 µg/mL) produced the most consistent cytoprotective effect following exposure to 25 and 50 µM H_2_O_2_ (Figure 4B) (H_2_O_2_ main F = 70.31; Wg-1 main F = 9.890; Interaction F = 7.48, *p* = 0.0003). However, no significant protection was observed at the higher H_2_O_2_ concentration tested, indicating that the protective effect was limited to a mild oxidative challenge. Since Wg-1 contained a minor co-occurring diterpenoid, the contribution of this constituent to the observed cytoprotective effect cannot be entirely excluded. *ent*-kaurenoic acid has previously demonstrated antioxidant and anti-inflammatory activity, reduced ROS and reactive nitrogen species production while enhancing cellular antioxidant defenses [45]. Proposed mechanisms include activation of the transcription factor Nrf2, demonstrated for *ent*-kaur-16-en-19-oic acid from *Aralia continentalis* [36] and downregulation of iNOS and the gp91phox subunit of NADPH oxidase, both implicated in free-radical generation. As the Nrf2 activation and downstream antioxidant responses were not evaluated in the present study, their involvement in Wg-1-mediated protection remains to be established. Furthermore, because Wg-1 exhibited a narrow biocompatibility range (Figure 4B), its concentration-dependent cytotoxic and cytoprotective effects require further investigation.

In contrast, pretreatment with Wg-2 (10 µg/mL) did not significantly prevent a reduction in cell viability at any of the H_2_O_2_ concentrations tested (Figure 4C) (H_2_O_2_ main F = 35.50; Wg-2 main F = 3.7; Interaction F = 0.2774, *p* = 0.91). The ortho-hydroxy-carbonyl motif is a classic transition metal (Fe^2+^/Cu^+^) chelator, sharing the same coordination geometry found in flavonoids and salicylates. The expected mechanism of protection against H_2_O_2_ involves the chemical suppression of the Fenton reaction, specifically, sequestering the iron or copper that catalyzes the conversion of H_2_O_2_ into the hydroxyl radical, thereby acting as an indirect protective mechanism [46]. However, under our experimental conditions, Wg-2 neither protected against nor exacerbated H_2_O_2_-induced cellular damage.

Similarly, pretreatment with Wg-3 (10 µg/mL) did not significantly alter cell viability relative to the corresponding H_2_O_2_-treated controls at any of the H_2_O_2_ concentrations tested (Figure 4D) (H_2_O_2_ main F = 28.62; TE main F = 0.42; Interaction F = 0.86, *p* = 0.51). Wg-3 was evaluated for potential protective effects against H_2_O_2_-induced damage, given that it is the direct synthetic precursor of a prenylated chalcone structurally analogous to xanthohumol, a compound of the same family with documented protective activity against oxidative damage in cells via Nrf2/HO-1 activation [47]. However, unlike xanthohumol, this acetophenone lacks the α,β-unsaturated enone (Michael acceptor) that activates the Keap1/Nrf2 pathway and possesses only a single phenolic hydroxyl group, lacking the ortho-dihydroxy (catechol) system required for efficient phenoxyl radical stabilization [48], consistent with the absence of protective activity observed.

### 2.5. Effects of TE and Selected Metabolites Against H_2_O_2_-Induced Intracellular ROS Levels

The DCFH-DA assay was used to kinetically monitor changes in intracellular probe oxidation in H_2_O_2_-exposed SH-SY5Y over a 6 h period, employing an oxidant-sensitive fluorescence probe. The 6 h monitoring period captures both the acute oxidant challenge and subsequent cellular responses that may influence DCF fluorescence, including H_2_O_2_ metabolism by catalase and glutathione peroxidase, as well as potential ROS and other oxidant species generation associated with cellular or compound metabolism.

**Figure 5:**
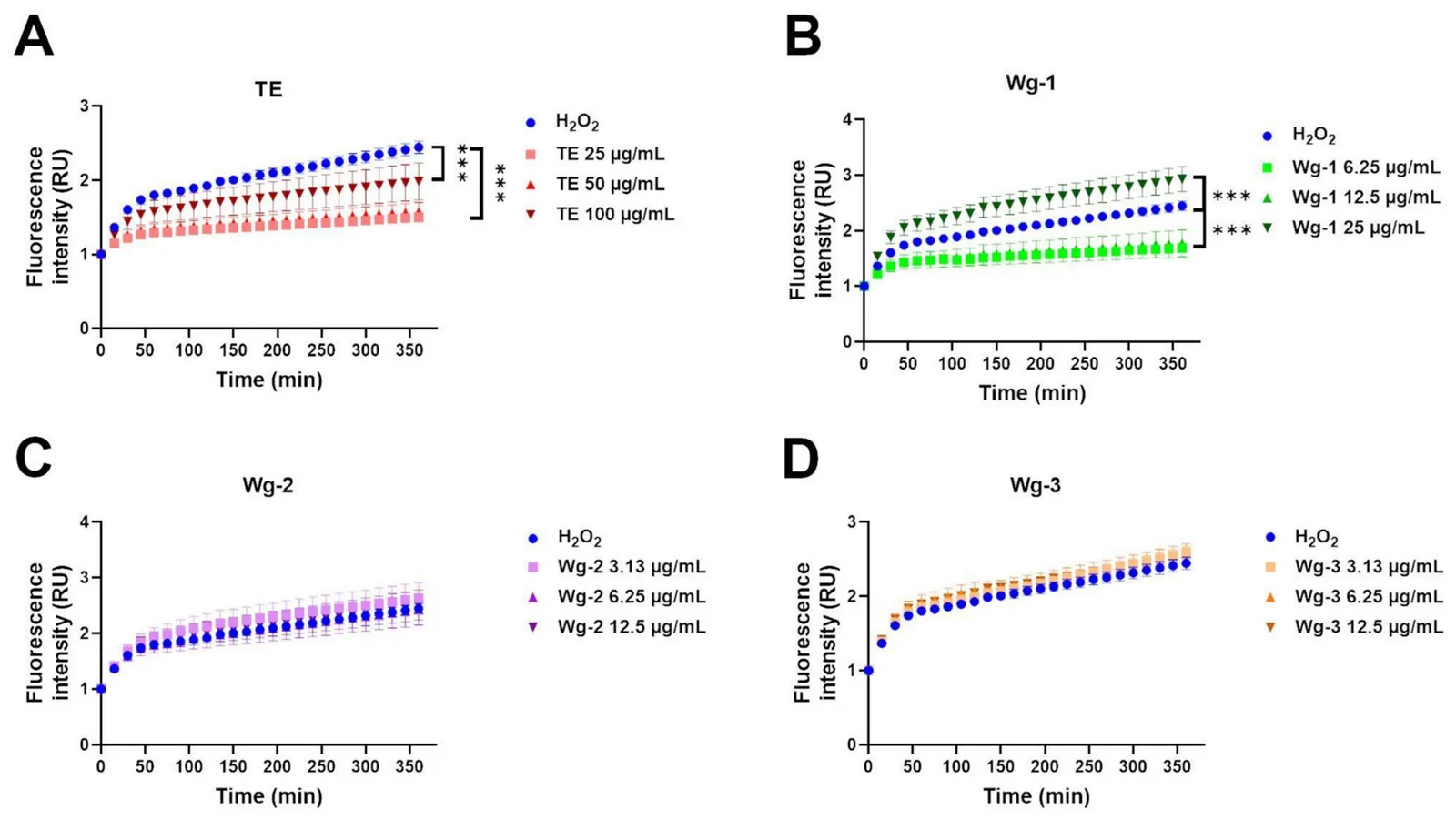
Intracellular ROS (DCFH-DA, 6 h kinetic with 50 µM H_2_O_2_) in SH-SY5Y cells treated with (**A**) 25, 50, and 100 µg/mL of TE, (**B**) 6.25, 12.5, and 25 µg/mL of Wg-1, (**C**) 3.13, 6.25, and 12.5 of Wg-2, and (**D**) Wg-3. Relative DCF fluorescence is shown as mean ± SEM; the 6 h endpoint was compared with the H_2_O_2_ control. Data corresponds to three independent biological replicates (*n* = 3). Statistical significance was determined by two-way ANOVA at each time point throughout the course of the experiment. Differences compared with the values of the respective control group are indicated on each graph by *** *p* < 0.001.

Pretreatment with TE significantly reduced the DCF signal at concentrations of 25, 50, and 100 μg/mL compared with the corresponding H_2_O_2_-treated control (Figure 5A) (Time main F = 12.81; TE main F = 175.9; Interaction F = 0.82, *p* = 0.82). Notably, TE attenuated H_2_O_2_-induced intracellular probe oxidation at 25 and 50 μg/mL, whereas cytoprotection was evaluated only at 100 μg/mL. Therefore, whether the reduction in DCF fluorescence observed at these lower concentrations is accompanied by preservation of cell viability remains to be determined. At 100 μg/mL, the reduction in DCF signal was accompanied by significant protection against H_2_O_2_-induced loss of viability (Figure 4A), supporting an association between modulation of the intracellular pro-oxidant environment and cytoprotection at this concentration.

Regarding the isolated metabolites, pretreatment with Wg-1 at 6.25 and 12.5 μg/mL reduced DCF fluorescence relative to the corresponding H_2_O_2_-treated control. In contrast, pretreatment with 25 μg/mL evoked an increased DCF fluorescence (Figure 5B) (Time main F = 16.45; Wg-1 main F = 230.9; Interaction F = 1.05, *p* = 0.38), indicating higher intracellular oxidation of the probe. Thus, the effect of Wg-1 on the oxidant-sensitive fluorescence probe was concentration-dependent but non-monotonic. At 6.25 μg/mL, Wg-1 attenuated the H_2_O_2_-induced increase in DCF fluorescence without reducing cell viability. This observation is broadly consistent with the cytoprotective effects observed with 10 μg/mL Wg-1 against milder H_2_O_2_ challenges (Figure 4B). However, Wg-1 produced a modest reduction in basal cell viability at 12.5 μg/mL (Figure 4B); therefore, the lower DCF signal at this concentration should be interpreted cautiously, as it may be partially influenced by changes in the number or metabolic state of viable cells. Notably, at 25 μg/mL, DCF fluorescence increased despite an approximately 50% reduction in cell viability (Figure 3B), suggesting increased probe oxidation under conditions of substantial cellular toxicity.

Previous studies have reported that *ent*-kaurenoic acid promotes Nrf2 nuclear accumulation and transcriptional activity in cellular models and exerts Nrf2-protective effects in an experimental model of inflammation [36]. Thus, Nrf2 activation could represent one possible mechanism contributing to the reduction in oxidant-sensitive fluorescence and cytoprotection observed at lower Wg-1 concentrations. However, this pathway was not assessed in the present study, and the published literature did not demonstrate direct binding of *ent*-kaurenoic acid to Keap1 or covalent modifications of its cysteine residues [36,49]. From a structural perspective, *ent*-kaurenoic acid lacks the phenolic hydroxyl groups commonly associated with hydrogen-atom donation and does not contain the α,β-unsaturated carbonyl system responsible for the electrophilic activity of certain oxidized *ent*-kaurane derivatives. Its carboxylic acid group is not a Michael acceptor. Consequently, neither direct radical scavenging nor covalent Keap1 modification can be inferred from its structure. Therefore, the mechanism underlying these effects for Wg-1 remains to be determined.

Pretreatment with Wg-2 or Wg-3 did not significantly alter H_2_O_2_-induced DCF fluorescence relative to the corresponding H_2_O_2_-treated controls at any time points evaluated (Figure 5C,D) (Time main F = 13.52; Wg-2 main F = 4.39; Interaction F = 0.02, *p* = 0.99; Time main F = 72.98; Wg-3 main F = 8.42; Interaction F = 0.06, *p* = 0.99). All Wg-2 concentrations tested in this assay were non-toxic in the cell viability assay. However, Wg-3 at 12.5 μg/mL reduced basal cell viability to approximately 60% of the control value. The absence of a significant change in total DCF fluorescence at this concentration should be interpreted with caution. Differences in the metabolic state and the number of viable cells could partly influence the measured signal. These results are consistent with the viability assays, in which neither compound protected against nor exacerbated H_2_O_2_-induced cellular damage under the experimental conditions tested. Nevertheless, the absence of an effect on DCF fluorescence does not exclude antioxidant activity under other concentrations, exposure periods, cellular models, or experimental systems. Antioxidant and cytotoxic activities have been reported for structurally diverse benzofuran derivatives [50]. However, these properties depend on the complete molecular structure and cannot be attributed to the benzofuran moiety alone. Accordingly, the lack of activity of Wg-2 in our assays neither demonstrates the absence of intrinsic antioxidant properties nor supports a cytotoxic mechanism, despite different benzofuran derivative analogues having shown to be a cytotoxic agent [51,52,53].

Similarly, the prenyl substituent of Wg-3 may influence its lipophilicity, cellular uptake, and subcellular distribution, but the direction and biological consequences of these effects cannot be predicted from structure alone. Counterintuitively, this often enhances activity in in vitro chemical assays in the stabilization of the phenoxyl radical by the olefinic double bond [54]. Nevertheless, in living cells, this excess lipophilicity can sequester the molecule within membranes rather than in the cytosol or mitochondria, where ROS are generated and measured. Cellular uptake or localization studies would be required to establish such a relationship.

### 2.6. Integrated Perspective and Future Directions

When the biocompatibility, cytoprotection, and DCF fluorescence data are integrated, Wg-1 displayed the most favorable overall profile. TE exhibited a broad biocompatibility window, protected SH-SY5Y cells against H_2_O_2_ at different concentrations, and attenuated H_2_O_2_-induced intracellular probe oxidation. Future experiments should evaluate whether cytoprotection is preserved at lower TE concentrations (25 and 50 μg/mL), at which reduced DCF fluorescence was observed after H_2_O_2_ exposure. The effect of TE may arise from one or several constituents. Although additive, synergistic, and antagonistic interactions have been described in other phytochemical mixtures, such interactions were not evaluated in the present study [55].

Among the isolated metabolites, Wg-1 showed the clearest biological activity. Pretreatment with 10 μg/mL Wg-1 protected cell viability against milder H_2_O_2_ challenges, whereas 6.25 and 12.5 μg/mL attenuated DCF fluorescence increase induced by H_2_O_2_. However, its relatively narrow biocompatibility range and the absence of a significant effect on DCF signal at 25 μg/mL indicate that its activity was restricted to lower concentrations, which is a common phenomenon when using isolated natural products [56,57,58].

Previous reports on Nrf2 activation by *ent*-kaurenoic acid provide a plausible context for these findings [36]. Although reproducing this previously described response in SH-SY5Y cells would provide evidence of its occurrence in a neuronal cellular context, a more informative objective would be to determine whether Nrf2 contributes functionally to the cytoprotective effect of Wg-1 and TE. This could be addressed by evaluating Nrf2 nuclear accumulation or ARE-dependent transcription and downstream targets such as HO-1, NQO1, and GCLC, followed by loss-of-function approaches.

The contribution of oxidative processes to a broad range of pathological conditions also provides a rationale for evaluating TE and Wg-1 activity in disease-relevant models. Given the neuronal origin of SH-SY5Y used here, neurodegenerative diseases represent one possible context for such studies, including differentiated neurons, co-culture systems, or neurodegenerative animal models [59,60].

Further characterization of TE is also warranted because its broader activity profile cannot be explained solely by three metabolites evaluated. Bioactivity-guided fractionation and testing of additional isolated constituents could identify other metabolites contributing to its cytoprotective or redox-modulating effects. Computational approaches could complement this experimental strategy by using the newly characterized metabolite profile to predict potential molecular targets and biological pathways and to prioritize compounds for subsequent evaluation and experimental validation.

## 3. Materials and Methods

### 3.1. Bioethical and Safety Methods

All the experimental procedures were carried out in accordance with the guidelines of the Agencia Nacional de Investigación y Desarrollo (ANID) and approved by the “Comité de Investigación Científica de la Universidad de Antofagasta” (No. 243; approved in 2019).

### 3.2. Collection and Botanical Characterization

The aerial parts of *Werneria glaberrima* (branches, stems, leaves, and flowers) were collected from its natural habitat near El Tatio in the Antofagasta Region, Chile (22°20′35.4″ S; 68°01′25.9″ W; at an altitude of 4273 m a.s.l.). Selective collection was carried out to minimize the impact on the area and ensure the survival and reproduction of the species. The samples were identified by Dr. Roberto Rodríguez (R.I.P.) of the Department of Botany, University of Concepción, Chile. Representative specimens were deposited in the herbarium of the University of Concepción, voucher number CONC 185.346, and a control for the herbarium of the Biological Chemistry Laboratory of the Antofagasta Institute (IA) of the University of Antofagasta, under No. 007-2022.

### 3.3. Preparation of Plant Extracts and Metabolites

#### 3.3.1. Preparation of Hydroalcoholic Extract (HAE-Wg)

The collected specimens were dried in the shade at room temperature and then finely ground using a mechanical mill. Approximately 1.00 kg of dried and ground plant material was placed in a cotton bag and left to stand for 48 h with 4 L of an EtOH:H_2_O (1:1) mixture. The resulting solution was then filtered using Whatman No. 4 filter paper and concentrated in a rotary evaporator to one-quarter of the initial volume. This procedure was repeated until the resulting solution was colorless. A portion of the concentrate was freeze-dried using a Labconco FreeZone 4.5 freezer and stored at 4 °C until use (UHPLC analysis, biological tests, and laboratory control). The remaining, non-freeze-dried portion was used to obtain the extracts by liquid–liquid partitioning.

#### 3.3.2. Obtaining Extracts by Liquid–Liquid Partitioning

The non-lyophilized hydroalcoholic concentrate was resuspended in 2 L of an 8:2 EtOH:H_2_O mixture and sequentially extracted with 2 L of n-hexane, dichloromethane (DCM), and finally ethyl acetate (EtOAc). Each organic solution was concentrated on a rotary evaporator to obtain a dry extract. All extracts were stored at 4 °C until used for the separation and purification of secondary metabolites.

#### 3.3.3. Separation and Purification of Metabolites

Metabolite separation was performed via gravity chromatography using silica gel as the stationary phase. To this end, 10 g of the *W. glaberrima* DCM extract were impregnated onto 15 g of silica gel through homogeneous mixing and loaded onto a column containing 300 g of silica. An elution system with a gradient of increasing polarity using mixtures of *n*-hexane and EtOAc (9:1 to 1:9) was employed. Fifty fractions were collected and subsequently pooled based on TLC analysis into six main fractions: A (3–4), B (5–10), C (11–16), D (17–25), E (26–48), and F (49–50). A crystalline precipitate was observed in fraction B; this was washed with n-hexane, yielding 212 mg of the pure compound Wg-2. The remaining supernatant from fraction B was further purified on silica gel using isocratic elution with *n*-hexane:EtOAc (7:3), yielding 127 mg of product Wg-3. Finally, fraction D was fractionated on a Sephadex LH-20 column using a chloroform:methanol (1:1) mixture as the eluent, resulting in a total of 25 fractions. Fractions 10–18 showed the presence of crystals; consequently, they were pooled according to their TLC profiles and recrystallized from cold n-hexane. The resulting crystals were dried and weighed (237 mg), yielding the product designated as Wg-1. ^1^H-NMR, ^13^C-NMR, HSQC/HMQC and HMBC analyses of each metabolite were performed using a Bruker Avance 400 MHz spectrometer. Infrared spectra were recorded on KBr pellets using a Nicolet Avatar 330 FT-IR instrument. The spectra were processed and analyzed using Mnova 17.0.1 software. The spectra of each metabolite are shown in the Supplementary Information (Figures S2–S16). The purity of the isolated compounds was assessed qualitatively by ^1^H/^13^C NMR. Wg-2 and Wg-3 showed no detectable impurity signals, whereas Wg-1 contained a minor co-occurring diterpenoid detectable in its NMR spectra (Figures S2 and S3).

### 3.4. UHPLC-ESI-Q Orbitrap-MS Analyses

Secondary metabolites from the *Werneria glaberrima* Phil. EHA extracts were profiled using a UHPLC Ultimate 3000 RS system equipped with Chromeleon 6.8 software (Dionex GmbH, Idstein, Germany) coupled to a Q Orbitrap mass spectrometer operating with Xcalibur 3.2 software. Chromatographic separation was achieved on an Acclaim Thermo C18 column (150 mm × 4.6 mm, 5 µm, 80 Å) at a flow rate of 0.90 mL/min. The mobile phase consisted of (A) 0.1% formic acid in water and (B) 0.1% formic acid in acetonitrile. The gradient elution program was as follows: 1% B (0–2 min), 1–5% B (2–3 min), 5% B (3–5 min), 5–10% B (5–8 min), 10–30% B (8–30 min), 30–95% B (31–38 min), and 1% B (39–50 min). For sample preparation, 5 mg of the extract was dissolved in 2 mL of methanol and filtered through a polytetrafluoroethylene (PTFE) membrane; the injection volume was 15 µL. Extract samples and methanol standards were maintained at 15 °C in the autosampler.

Mass spectrometry was performed using a heated electrospray ionization (HESI II) probe. Based on previously reported parameters, the sheath and auxiliary gas flows were set to 75 and 25 units, respectively. The capillary temperature was maintained at 420 °C, the auxiliary gas heater at 520 °C, the S-lens RF level at 32, and the spray voltage at 2500 V for negative ionization mode (ESI−). Full-scan MS data were acquired in both positive and negative polarities over an *m*/*z* range of 70–1000 at a resolution of 70,000 FWHM (at *m*/*z* 200). The automatic gain control (AGC) target was set to 3 × 10^6^ with a maximum injection time of 200 ms. High-purity nitrogen (>99.99%) supplied by a Genius NM32LA generator (Peak Scientific, Billerica, MA, USA) served as the collision and damping gas. Orbitrap mass calibration was executed twice daily in both ionization modes to guarantee a mass accuracy within 5 ppm. Structural characterization of bioactive compounds was established using HR full MS data, fragmentation patterns, and literature comparison.

### 3.5. Total Phenolic Content (TPC) Estimation

The TPC was based on the 96-well microplate Folin–Ciocalteu method (Sigma-Aldrich, St. Louis, MO, USA). A total of 20 μL of the diluted extract (500 μg/mL) was mixed with 100 μL of 10% (*vol*/*vol*) of Folin–Ciocalteu reagent and shaken. The mixture was left for 5 min, and then 80 μL of sodium carbonate solution (700 mM (7.5%)) was added. The mixture was shaken for 1 min. After 60 min at room temperature, the absorbance was measured at 765 nm using the microplate reader. The absorbance of the same reaction with water instead of the extract or standard was subtracted from the absorbance of the reaction with the sample. Gallic acid dilutions (0–1000 μg/mL) were used as standards for calibration. The results were expressed as mg of gallic acid equivalent per gram of dry extract. All experiments were done in triplicate (*n* = 3) [61].

### 3.6. Total Flavonoid Content (TFC) Estimation

The total flavonoid contents were determined by the aluminum chloride colorimetric method (Sigma-Aldrich, St. Louis, MO, USA). 50 μL of the extract (final concentration 500 μg/mL in MeOH) was pipetted into a microplate to which was added 50 μL of methanol, 20 μL of 10% aluminum chloride, and 20 μL of 1 M potassium acetate aqueous solution. A yellow color indicated the presence of flavonoids. The final sample solution in the microplate was left to incubate for 30 min at room temperature, and the absorbance of the solution was read at 510 nm. Analyses were carried out in triplicate (*n* = 3), and the results were expressed as mg quercetin equivalent per gram of dry extract by comparison with the standard curve [62].

### 3.7. Ferric Reducing Antioxidant Power (FRAP) Assay

The FRAP assay was carried out by the following method. The FRAP reagent included 300 mM acetate buffer pH 3.6, 10 mM of 2,4,6-tris-(2-pyridyl)-s-triazine (TPTZ) solution in 40 mM hydrochloric acid, and 20 mM FeCl_3_·6H_2_O aqueous solution in the ratio of 10:1:1 (*v*/*v*). The extracts were prepared at a final concentration of 500 μg/mL. Extract solution (30 μL) was mixed with 170 μL of freshly prepared FRAP reagent solution and incubated at 37 °C for 30 min. The absorbance of the solutions was measured at 593 nm. Trolox was used as the standard solution to draw the calibration curve in a concentration range of 0–500 μg/mL. The FRAP results were expressed as mg of Trolox equivalent per gram of dry extract. All experiments were done in triplicate (*n* = 3) [63].

### 3.8. Metal Chelating Assay

Ferrous ion chelation was evaluated by measuring the reduction in Fe^2+^–ferrozine complex formation following a previously published protocol with minor modifications [64]. For each reaction, 50 μL of extract (0–1000 μg/mL) was combined with 10 μL of 2 mM FeCl_2_. The reaction was initiated by adding 20 μL of 5 mM ferrozine, followed by 70 μL of methanol. After 10 min at room temperature, absorbance was recorded at 562 nm. EDTA was assayed over the same concentration range as a reference chelator. The results were expressed as IC_50_ (concentration of extract or standard in μg/mL required to inhibit 50% of complex formation). Analyses were carried out in triplicate (*n* = 3).

### 3.9. DPPH Radical Scavenging Activity Assay

Quantitative measurement of radical scavenging properties of the extract was carried out by the following method. A 0.2 mM solution of 1,1-diphenyl-2-picrylhydrazyl (DPPH) (Sigma-Aldrich, St. Louis, MO, USA) in methanol was prepared, and 70 μL of this solution was added to 20 μL of extract (0–1000 μg/mL). Trolox (Fluka, Dorset, UK) in concentrations of 0–1000 μg/mL was used as a reference antioxidant. Discoloration of the reaction mixture was measured at 517 nm after incubation for 30 min. The results were expressed as IC_50_ (concentration of extract or standard in μg/mL required to inhibit 50% of DPPH radicals present in solution). Analyses were carried out in triplicate (*n* = 3) [65].

### 3.10. Determination of ABTS Radical-Scavenging Activity

The radical scavenging capacity of the samples for the ABTS radical cation was carried out by the following method. ABTS (Sigma-Aldrich, St. Louis, MO, USA) was generated by mixing a 7 mM stock solution of ABTS in PBS at pH 7.4 with 2.5 mM K_2_S_2_O_8_ (final concentration) followed by storage in the dark at room temperature for 16 h before use. The mixture was diluted with PBS to give an absorbance of 0.700 ± 0.02 units at 734 nm using a spectrophotometer. For each sample, diluted methanol solution of the sample (30 μL) was allowed to react with fresh ABTS solution (180 μL), and then the absorbance was measured 6 min after initial mixing. The blank was prepared from 180 μL of solution of the ABTS in PBS and 30 μL of PBS. Trolox was used as a standard, and the capacity of free radical scavenging was expressed by IC_50_ (μg/mL) values calculated to denote the concentration required to scavenge 50% of ABTS radicals. All measurements were performed in triplicate (*n* = 3). The results are expressed in mmol Trolox equivalent (TE)/g dry extract (TEAC) [66,67].

### 3.11. Cell Culture and Treatment of Extract and Metabolites

The human neuroblastoma SH-SY5Y cell line was maintained in DMEM supplemented with 10% fetal bovine serum (FBS), 1% GlutaMAX, and antibiotics (Thermo Fisher Scientific, Waltham, MA, USA) in an incubator at 37 °C, 5% CO_2_, and 90% humidity. Confluence was monitored every 48-h. Subculturing was performed at 60–80% confluence by PBS washing, trypsin dissociation (3 min, 37 °C), and resuspension in complete medium. For the assays, cells were seeded in 96-well plates at 30,000 cells/well in 100 µL. For the ROS assay, wells were pre-coated with poly-L-lysine (Thermo Fisher Scientific, Waltham, MA, USA) to improve cell adherence.

TE was dissolved in sterile water, and isolated metabolites in DMSO (Merck, Darmstadt, Germany) were prepared as stock solutions (10 mg/mL), which were diluted in culture medium immediately before use. The highest final concentration of DMSO was 1% (*v*/*v*). For biocompatibility, cells were exposed for 24 h to increasing concentrations of TE or metabolite (6.25–400 µg/mL). For cytoprotection, cells were pretreated for 24 h with TE (100 µg/mL) or metabolites (10 µg/mL). The treatment medium was then removed, cells were washed once with PBS, and fresh medium containing H_2_O_2_ (12.5–200 µM) without TE or metabolites was added for 2 h. Cells treated in parallel with the vehicle followed by H_2_O_2_ served as controls. Cell viability was assessed immediately after H_2_O_2_ exposure.

### 3.12. Cell Viability by Resazurin Reduction (Alamar Blue)

Viability was determined by the Alamar Blue assay (Thermo Fisher Scientific, Waltham, MA, USA), based on the resazurin-to-resorufin reduction, which was used according to the manufacturer’s instructions, measuring fluorescence at 530 nm excitation and 590 nm emission. Values were expressed as percentage viability relative to vehicle control.

### 3.13. Assessment of Intracellular Oxidation Using the H_2_DCF-DA Probe

The intracellular oxidative environment was measured with the 2′,7′-dichlorodihydrofluorescein diacetate (H_2_DCF-DA) probe (Thermo Fisher Scientific, Waltham, MA, USA) according to the manufacturer’s instructions, which is non-fluorescent and is hydrolyzed by intracellular esterases to H_2_DCF and oxidized by reactive species to the fluorescent product DCF (480 nm excitation, 528 nm emission). After treatments with TE or isolated metabolites, the medium was replaced with the 10 µM of H_2_DCF-DA probe in DMEM FluoroBrite (Thermo Fisher Scientific, Waltham, MA, USA) and incubated for 30 min to allow internalization. The probe solution was then removed, and cells were exposed to 50 µM H_2_O_2_ in FluoroBrite DMEM without TE or metabolites, and fluorescence (Ex 480/Em 528 nm) was recorded every 15 min for 6 h.

### 3.14. Statistical Analysis

Data are expressed as the mean ± standard error of the mean (SEM) of three independent biological replicates (*n* = 3). Normality of the ANOVA residuals was assessed by QQ plots and the D’Agostino-Pearson test. Biocompatibility and the cytoprotection concentration curve were analyzed by one-way ANOVA using Bonferroni’s correction as a post-hoc test. The pretreatment design against H_2_O_2_ and the oxidation of DCF level curves were analyzed by two-way ANOVA using Sidak as a post-hoc test. Statistical analyses were performed using GraphPad Prism 10.6.1 (GraphPad Software, Inc., San Diego, CA, USA). The value of *p* < 0.05 was considered significant.

### 3.15. Use of Artificial Intelligence (AI)-Assisted Technology

The author(s) confirms that they have used Artificial Intelligence (AI)-Assisted Technology for assisting in the writing of the manuscript and searching for scientific article references. Not for study design, data collection, analysis, or interpretation.

## 4. Conclusions

This study provides the first detailed phytochemical characterization of the total extract (TE) of the high-Andean medicinal plant *W. glaberrima* and initial in vitro evidence of its cytoprotection and potential antioxidant activity in H_2_O_2_-challenged SH-SY5Y cells. TE exhibited a broad biocompatibility range, protected against H_2_O_2_-induced loss of cell viability, and the pretreatment attenuated intracellular probe oxidation after H_2_O_2_ exposure. Moreover, three metabolites were isolated: Wg-1 (*ent*-kaurenoic acid), Wg-2 (benzofuran), and Wg-3 (acetophenone). Among them, Wg-1 displayed cytoprotection accompanied by the reduction in H_2_O_2_-induced DCF fluorescence in SH-SY5Y cells at lower concentrations. In contrast, Wg-2 and Wg-3 neither protected against nor exacerbated H_2_O_2_-induced loss of viability and did not modify DCF oxidation after H_2_O_2_ exposure at the concentration tested. Overall, these results preliminarily position *W. glaberrima* as a source of bioactive compounds with cytoprotective and antioxidant potential, providing a basis for identifying additional active constituents and evaluating TE and Wg-1 in more physiologically and oxidative-stress-associated pathology models.

## Figures and Tables

**Table 1:** Tentative annotation of metabolites in the total extract of *W. glaberrima* by UHPLC–ESI–QOrbitrap–MS.

Peak	Tentative Identification	[M-H]^−^	Retention Time (min)	Measured Mass (*m*/*z*)	Theoretical Mass (*m*/*z*)	Accuracy (ppm)	Metabolite Type	MS Ions (ppm)
1	Quinic acid	C_7_H_11_O_6_	1.47	191.05546	191.05501	2.35	Phenolic acid	-
2	Rhapontin	C_21_H_23_O_9_	1.56	419.13470	419.13465	0.11	Stilbene	271.05714, 269.0444, 255.06280
3	4,7,10,13-tetraoxahexadecanedioic acid	C_12_H_21_O_8_	8.71	293.12558	293.12762	2.5	Fatty acid	135.02740
4	Foliachinenoside H	C_17_H_29_O_12_	9.07	425.16828	425.16988	−3.6	Sugar	329.09259
5	Chlorogenic acid	C_16_H_17_O_9_	9.34	353.08923	353.08957	−0.96	Phenolic acid	191.05631
6	Kaempferol-3-O-rutinoside	C_27_H_29_O_15_	9.55	593.15338	593.15323	0.25	Flavone	285.04631
7	Asperuloside	C_18_H_21_O_11_	10.02	413.10770	413.10884	−2.75	Iridoid	191.03445, 175.0390
8	Neochlorogenic acid	C_16_H_17_O_9_	10.52	353.08936	353.08957	−0.59	Phenolic acid	191.05634
9	Cynarin	C_25_H_23_O_12_	11.76	515.12076	515.12025	0.99	Phenolic acid	191.05634, 135.04531
10	6-Methoxy-2-naphthylacetic acid	C_13_H_11_O_3_	11.89	215.07124	215.07127	−0.13	Naphthyl acid	149.9947; 119.0494
11	Salicortin	C_27_H_29_O_13_	12.02	561.16551	561.16649	−1.74	Phenolic acid	417.0336, 180.0565
12	Combretol	C_20_H_19_O_8_	12.48	387.10805	387.10878	−1.88	Flavone	353.89362, 317.91678, 275.97464, 165.02860, 239.05757
13	Kaempferol	C_15_H_9_O_6_	13.47	285.04055	285.04070	−0.52	Flavone	179.03503
14	3,4-Dimethoxycinnamic acid	C_11_H_11_O_4_	14.46	207.06534	207.06519	0.72	Phenolic acid	132.02253, 103.05172
15	Acetylsalicylsalicylic acid	C_16_H_11_O_6_	16.76	299.05528	299.05552	−0.80	Phenolic acid	255.82314
16	Tetracosanoic acid	C_24_H_47_O_2_	18.43	367.35880	367.35812	1.85	Fatty acid	311.16107
17	Atractyloside-A-14-O-Beta-D-Fructofuranoside	C_27_H_45_O_15_	19.12	609.27195	609.27127	1.11	Sugar	347.18796, 160.84140
18	Arachidic acid	C_20_H_39_O_2_	22.56	311.16814	311.16870	−1.79	Fatty acid	215.83909
19	Kazinol K	C_30_H_37_O_4_	23.75	461.26959	461.26864	2.07	Chromene	311.16103
20	*ent*-kaur-16-en-19-oic acid	C_20_H_29_O_2_	25.35	301.21766	301.21732	1.12	Terpene	258.23475
21	Unknown compound	C_20_H_29_O_3_	25.86	317.21153	317.21112	1.29	Terpene	265.14911

**Table 2:** In vitro antioxidant activity of hydroalcoholic extract of *Werneria glaberrima* Phil.

Hydroalcoholic Extract	Total Phenols ^a^	Total Flavonoids ^b^	FRAP ^c^	IC_50_ Chelating Activity ^d^	IC_50_ DPPH ^d^	IC_50_ ABTS ^d^
*W. glaberrima*	234.6 ± 11.4	115.6 ± 8.76	192.7 ± 15.6	116.7 ± 1.6	284.4 ± 14.5	342.4 ± 12.4
Trolox	--	--	--	--	8.75 ± 1.1	11.7 ± 1.8
EDTA	--	--	--	5.2 ± 0.79	--	--

All values were expressed as Means ± SEM (*n* = 3). ^a^ expressed in mg Gallic acid equivalent/g dry extract. ^b^ expressed in mg Quercetin equivalent/g dry extract. ^c^ expressed in mg Trolox equivalent/g dry extract. ^d^ expressed in µg/mL.

## Data Availability

The original contributions presented in this study are included in the article/Supplementary Materials. Further inquiries can be directed at the corresponding authors.

## Supplementary Materials

The following supporting information can be downloaded at: https://www.mdpi.com/article/10.3390/molecules31203765/s1, Figure S1: Distribution of tentatively annotated metabolites of *W. glaberrima* TE among the identified chemical classes, expressed as a percentage of the total number of annotated compounds; Figure S2: ^1^H-NMR spectrum of Wg-1 (*ent*-kaurenoic acid) (CDCl_3_, 400 MHz); Figure S3: ^13^C-NMR spectrum of Wg-1 (CDCl_3_, 101 MHz); Figure S4: HSQC spectrum of Wg-1 (CDCl_3_); Figure S5: HMBC spectrum of Wg-1 (CDCl_3_); Figure S6: FT-IR spectrum of Wg-1 (KBr); Figure S7: ^1^H-NMR spectrum of Wg-2 (1-[6-hydroxy-2-(prop-1-en-2-yl)-2,3-dihydro-1-benzofuran-5-yl]ethan-1-one) (CDCl_3_, 400 MHz); Figure S8: ^13^C-NMR spectrum of Wg-2 (CDCl_3_, 101 MHz); Figure S9: HSQC spectrum of Wg-2 (CDCl_3_); Figure S10: HMBC spectrum of Wg-2 (CDCl_3_); Figure S11: FT-IR spectrum of Wg-2 (KBr); Figure S12: ^1^H-NMR spectrum of Wg-3 (4-hydroxy-3-(3-methyl-2-butenyl)acetophenone) (CDCl_3_, 400 MHz); Figure S13: ^13^C-NMR spectrum of Wg-3 (CDCl_3_, 101 MHz); Figure S14: HMQC spectrum of Wg-3 (CDCl_3_); Figure S15: HMBC spectrum of Wg-3 (CDCl_3_); Figure S16: FT-IR spectrum of Wg-3 (KBr).
