# Supplementary material for: Phytochemical Composition, Cytoprotective and Antioxidant Activity of Werneria glaberrima Against H2O2-Induced Oxidative Stress in Human SH-SY5Y Neuroblastoma Cells
Source: Molecules. 2026 Oct 20;31(20):3765. doi: 10.3390/molecules31203765 (PMC13707604; doi:10.3390/molecules31203765)
Supplement: Supplementary file 1 [file molecules-31-03765-s001.zip › molecules-4567119-supplementary.pdf]

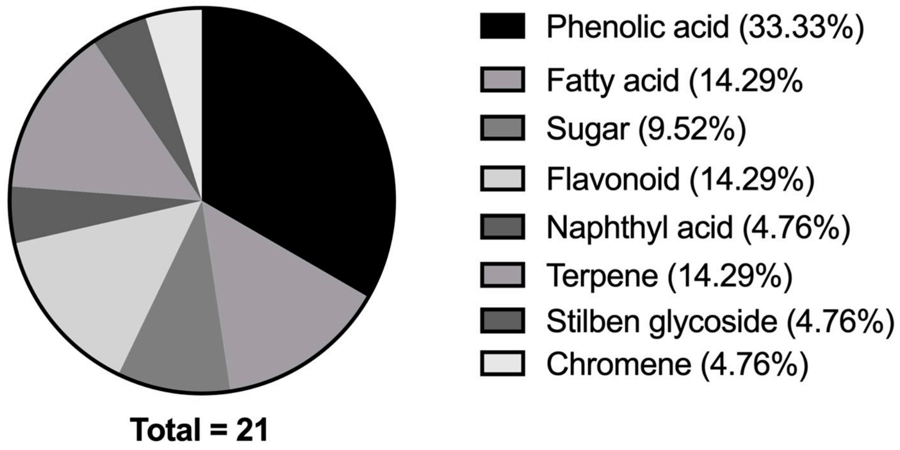

Figure S1. Distribution of tentatively annotated metabolites of *W. glaberrima* TE among the identified chemical classes, expressed as a percentage of the total number of annotated compounds.

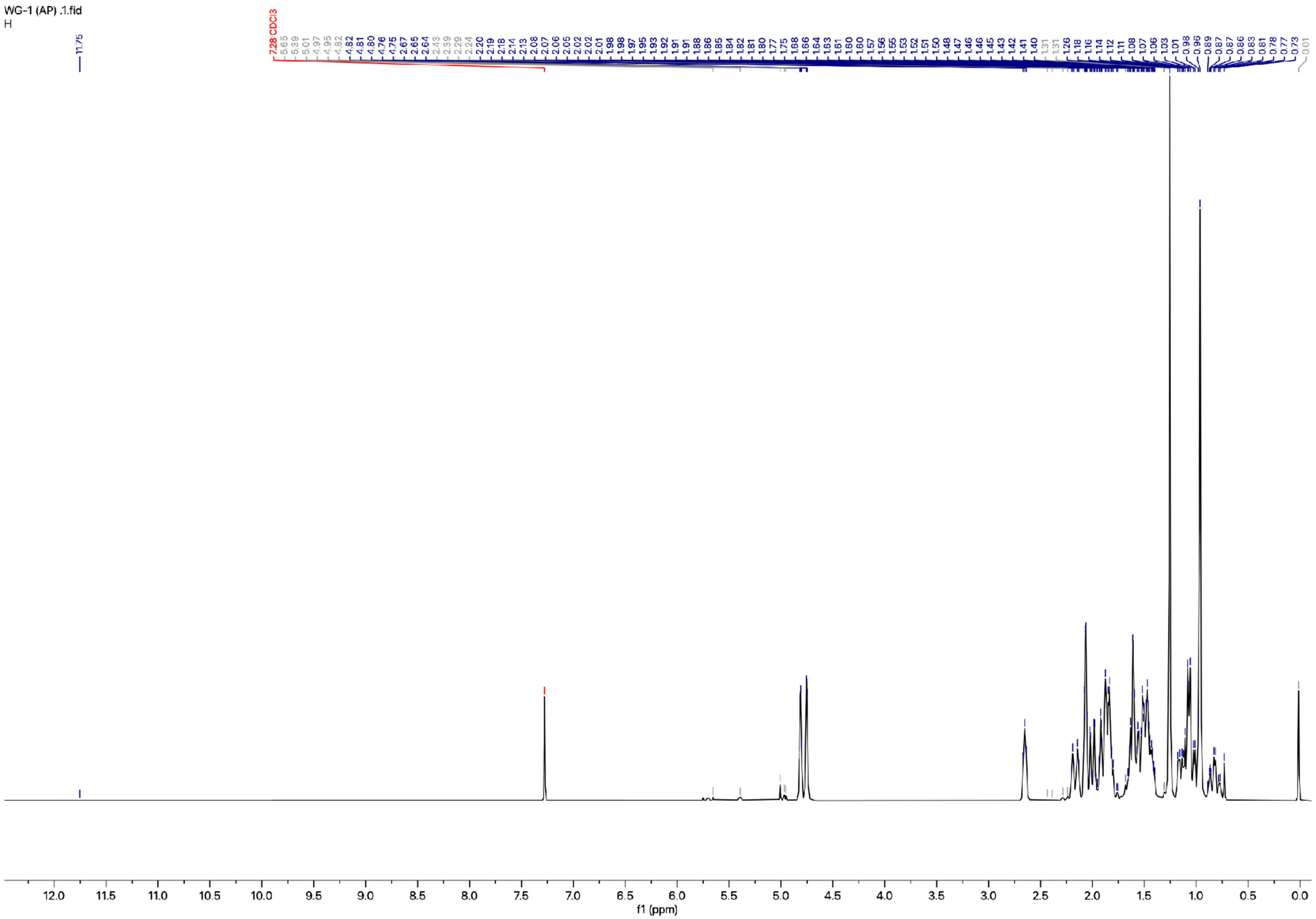

Figure S2. ^{1}H-NMR of *ent*-kaurenoic acid ($CDCl_3$, 400 MHz) δ 0.70 – 0.92 (m, 1H), 0.96 (s, 3H), 0.95 – 1.21 (m, 4H), 1.26 (s, 3H), 1.37 – 1.70 (m, 6H), 1.70 – 2.10 (m, 8H), 2.10 – 2.23 (m, 1H), 2.64 (d, *J* = 4.5 Hz, 1H), 4.72 – 4.85 (m, 2H), 11.75 (s, 1H).

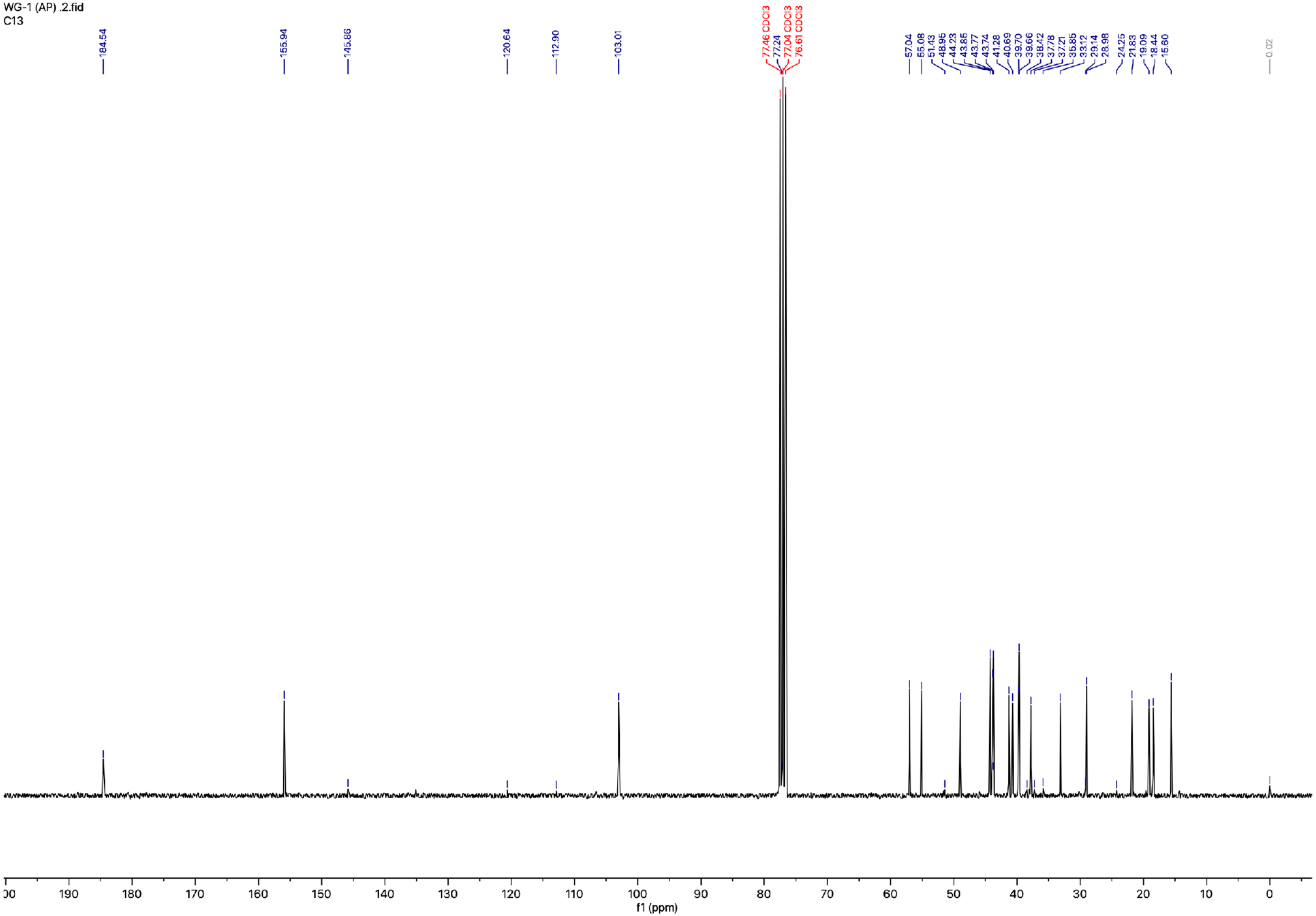

Figure S3. **^{13}C-NMR of *ent*-kaurenoic acid ($CDCl_3$, 101 MHz) δ 184.54, 155.94, 145.86, 120.64, 112.90, 103.01, 77.24, 57.04, 55.08, 51.43, 48.95, 44.23, 43.85, 43.77, 43.74, 41.28, 40.69, 39.70, 39.66, 38.42, 37.78, 37.21, 35.85, 33.12, 29.14, 28.98, 21.83, 19.09, 18.44, 15.60**

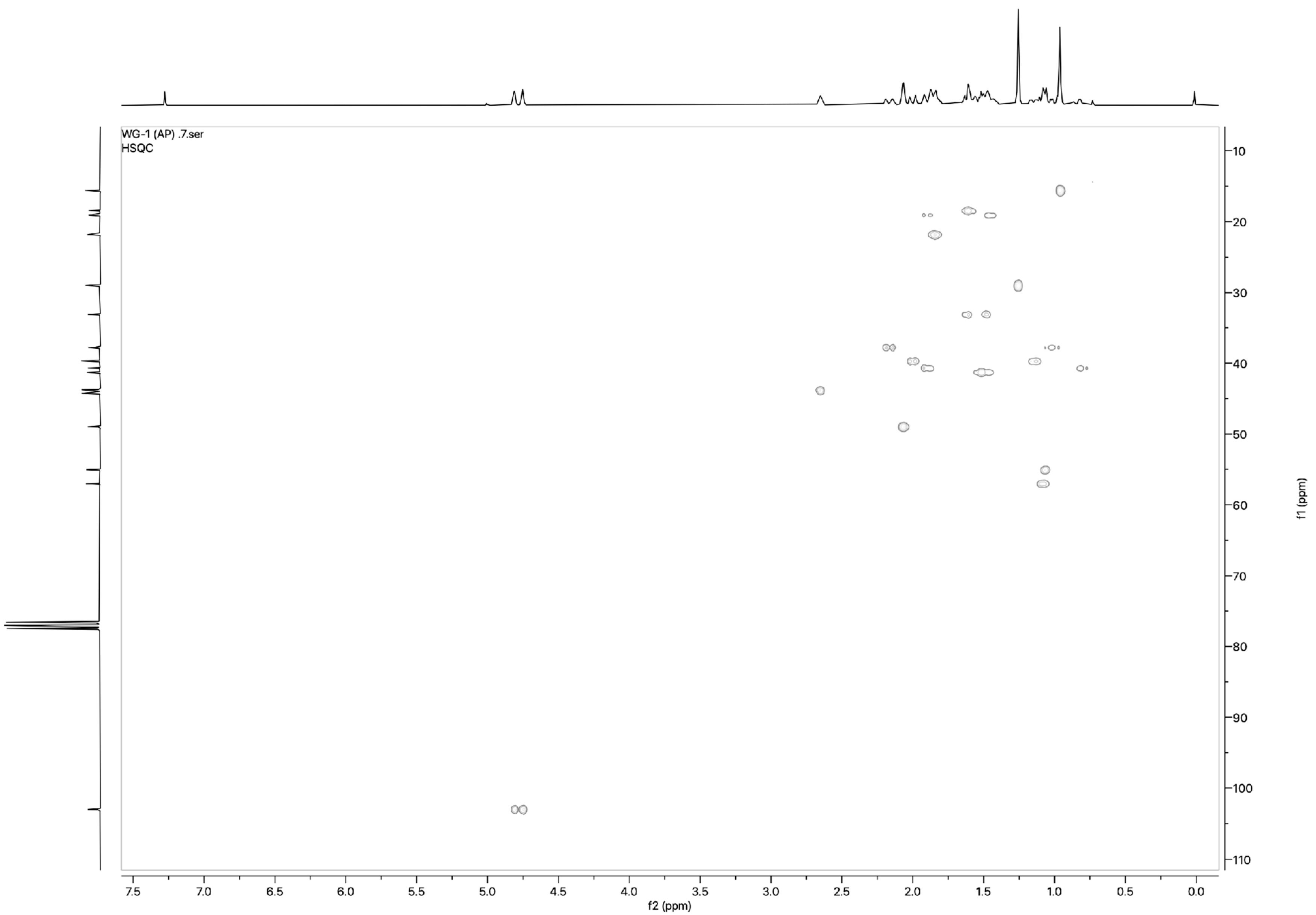

Figure S4. HSQC spectrum of *ent*-kaurenoic acid ($CDCl_3$)

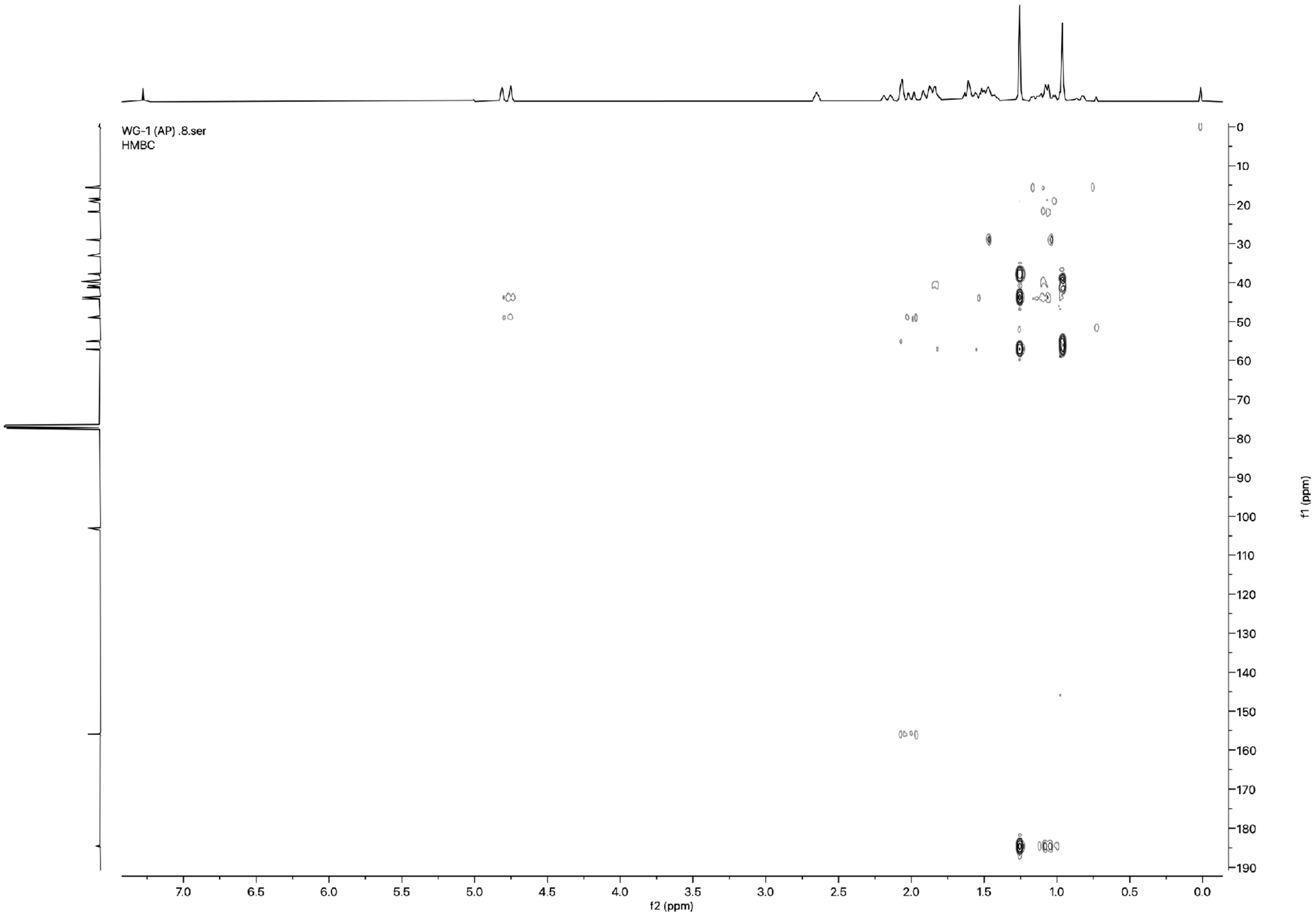

Figure S5. HMBC spectrum of *ent*-kaurenoic acid ($CDCl_3$)

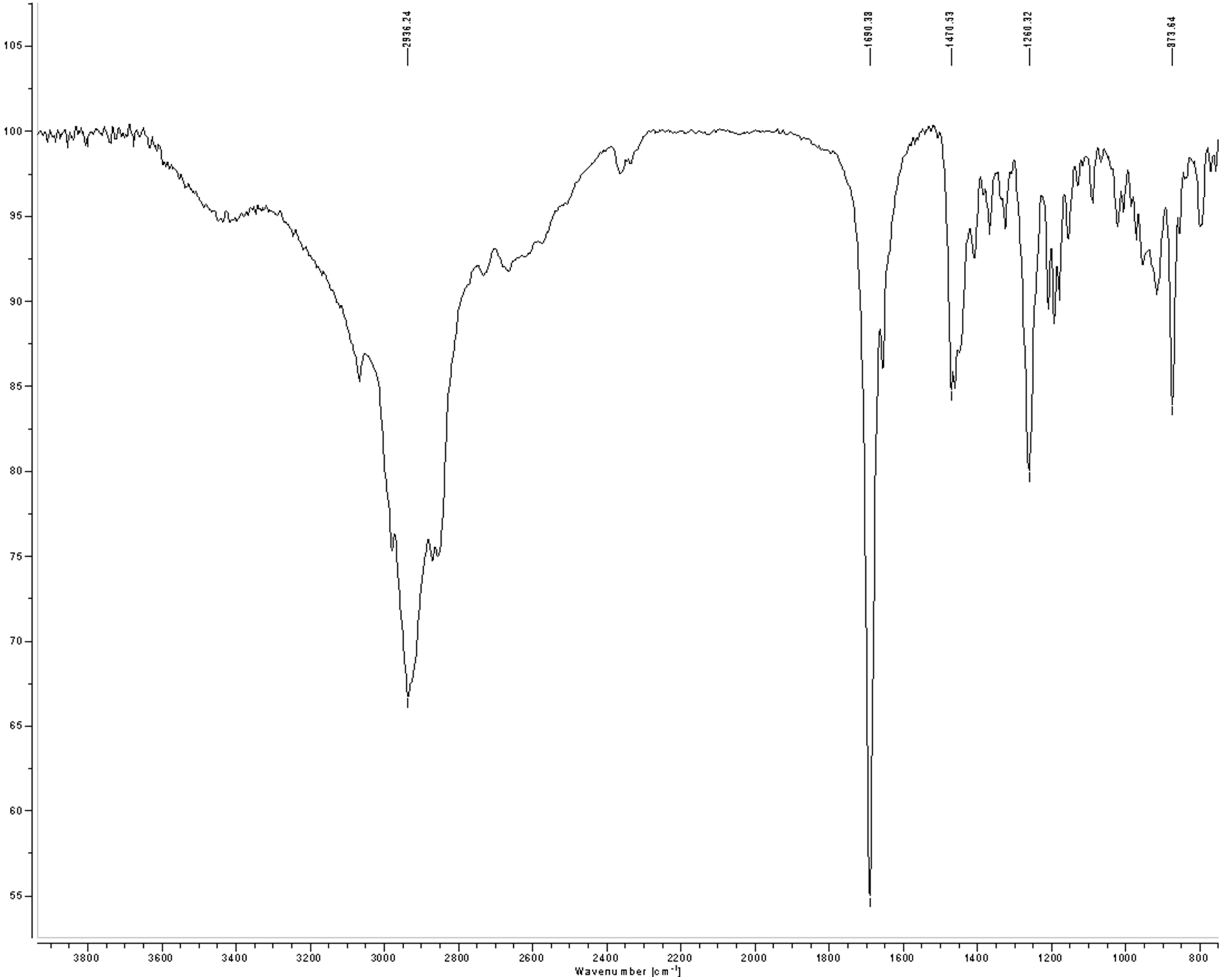

Figure S6. FT-IR Spectrum of *ent*-kaurenoic acid (KBr)

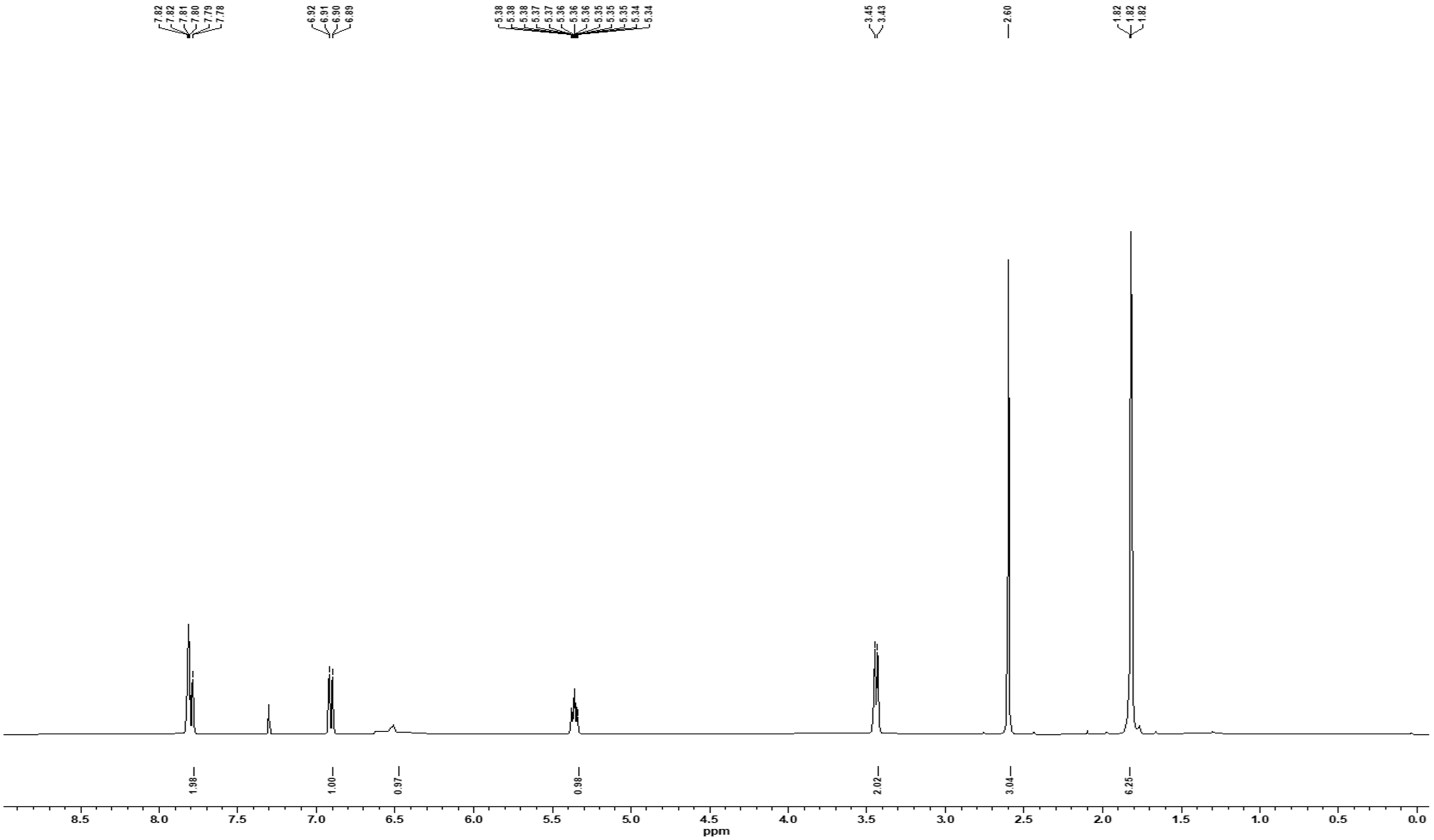

Figure S7. ¹H-NMR Spectrum of 4-Hydroxy-3-(3-methyl-2-butenyl)acetophenone ($CDCl_3$, 400 MHz) δ 1.82 (s, 6H), 2.60 (s, 3H), 3.44 (d, *J* = 7.3 Hz, 2H), 5.36 (t, *J* = 6.6 Hz, 1H), 6.27–6.69 (m, 1H), 6.91 (dd, *J* = 8.2, 2.9 Hz, 1H), 7.80 (dd, *J* = 10.5, 2.3 Hz, 2H).

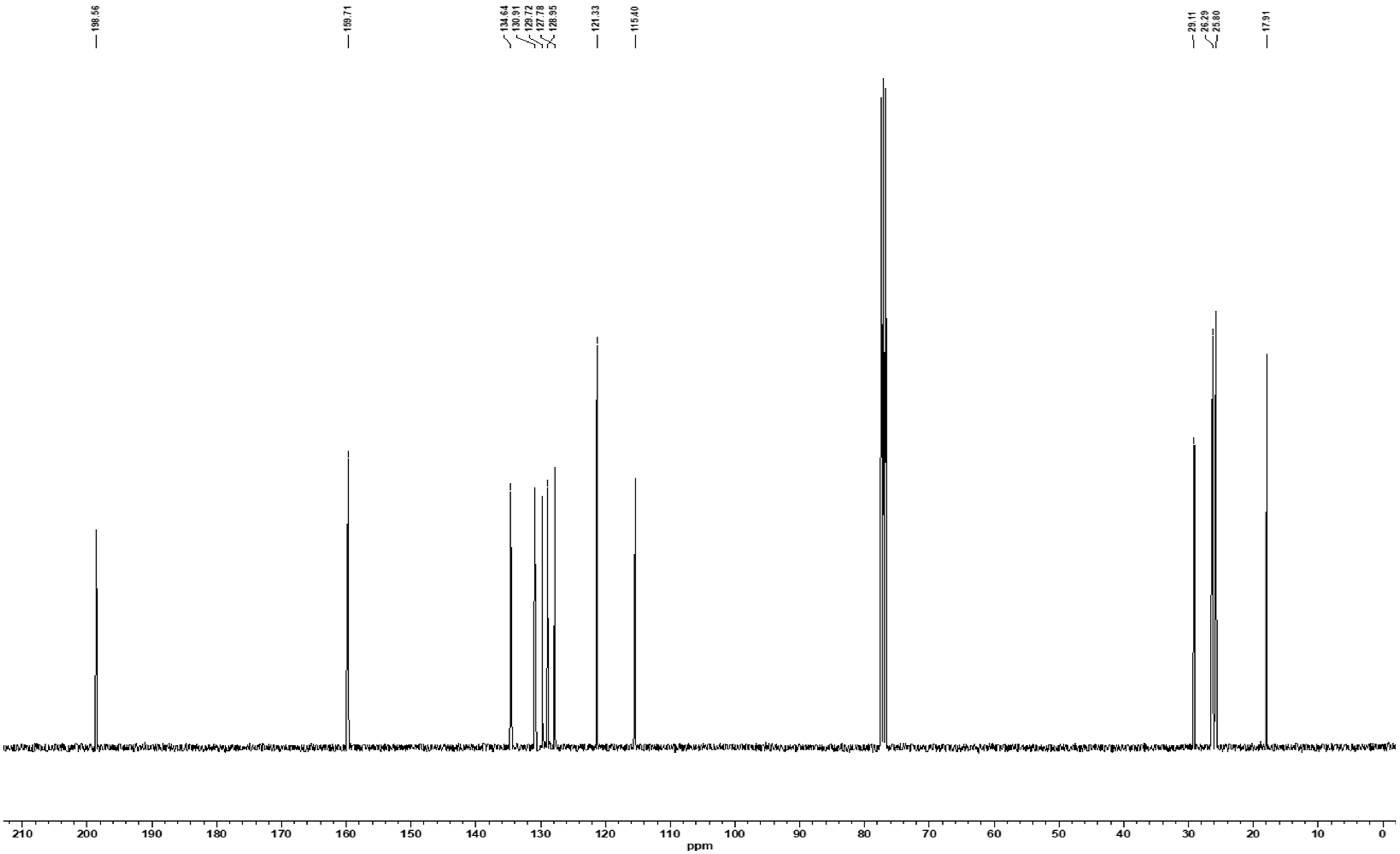

Figure S8. ^{13}C-NMR Spectrum of 4-Hydroxy-3-(3-methyl-2-butenyl)acetophenone ($CDCl_3$, 101 MHz) δ 198.56, 159.71, 134.64, 130.91, 129.72, 128.95, 127.78, 121.33, 115.40, 77.41, 77.09, 76.77, 29.11, 26.29, 25.80, 17.91.

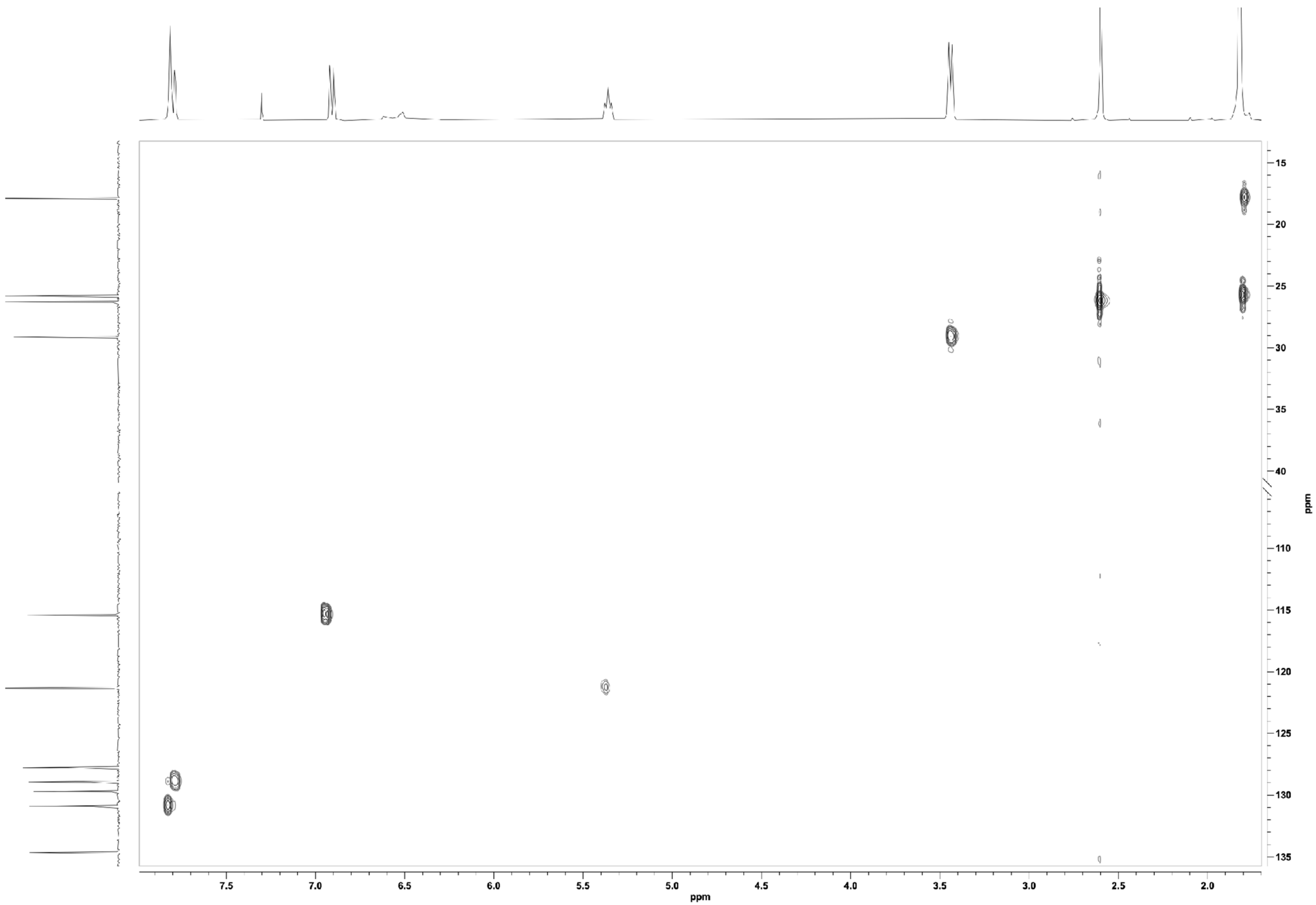

Figure S9. HMQC Spectrum of 4-Hydroxy-3-(3-methyl-2-butenyl)acetophenone

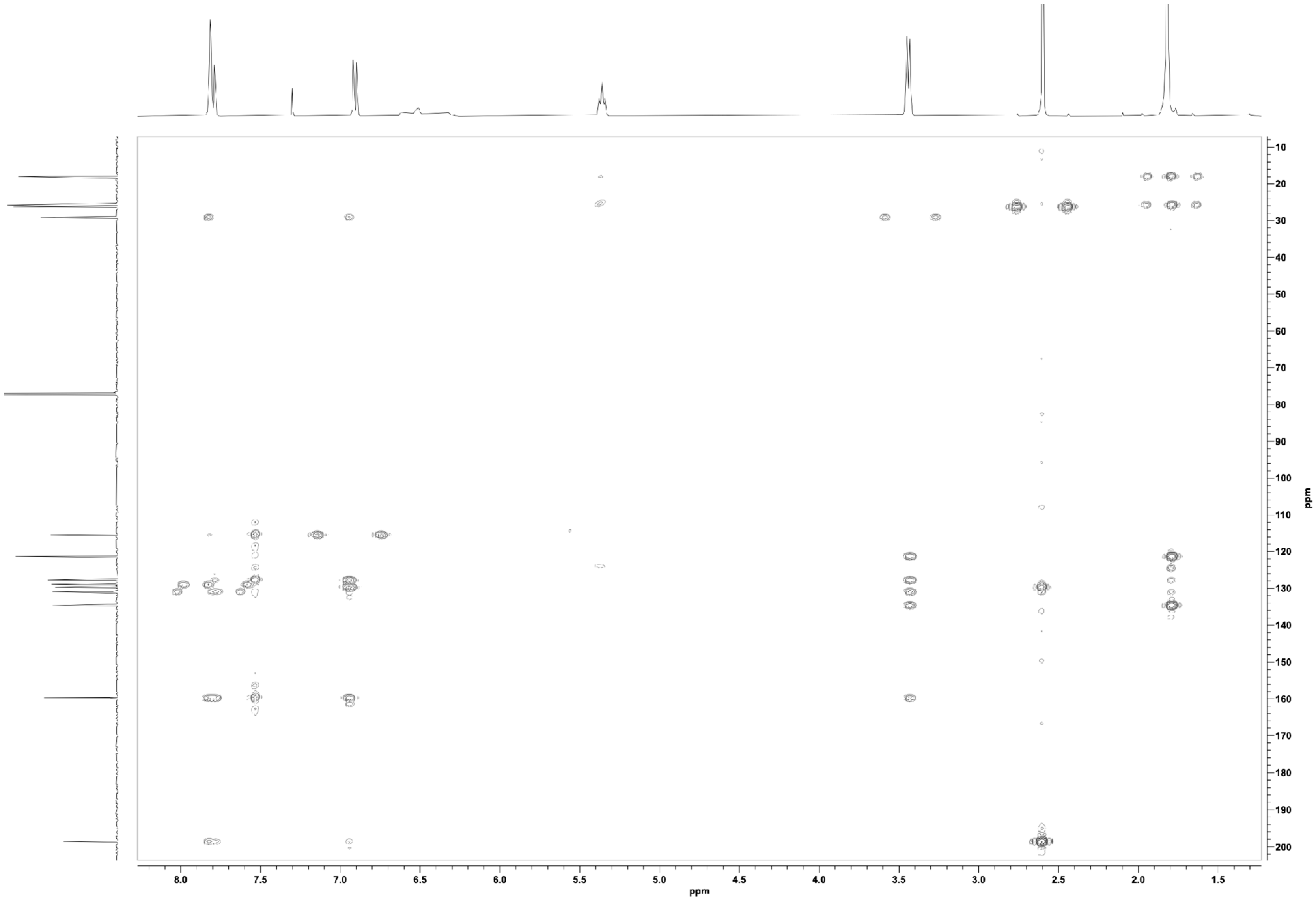

Figure S10. HMBC Spectrum of 4-Hydroxy-3-(3-methyl-2-butenyl)acetophenone

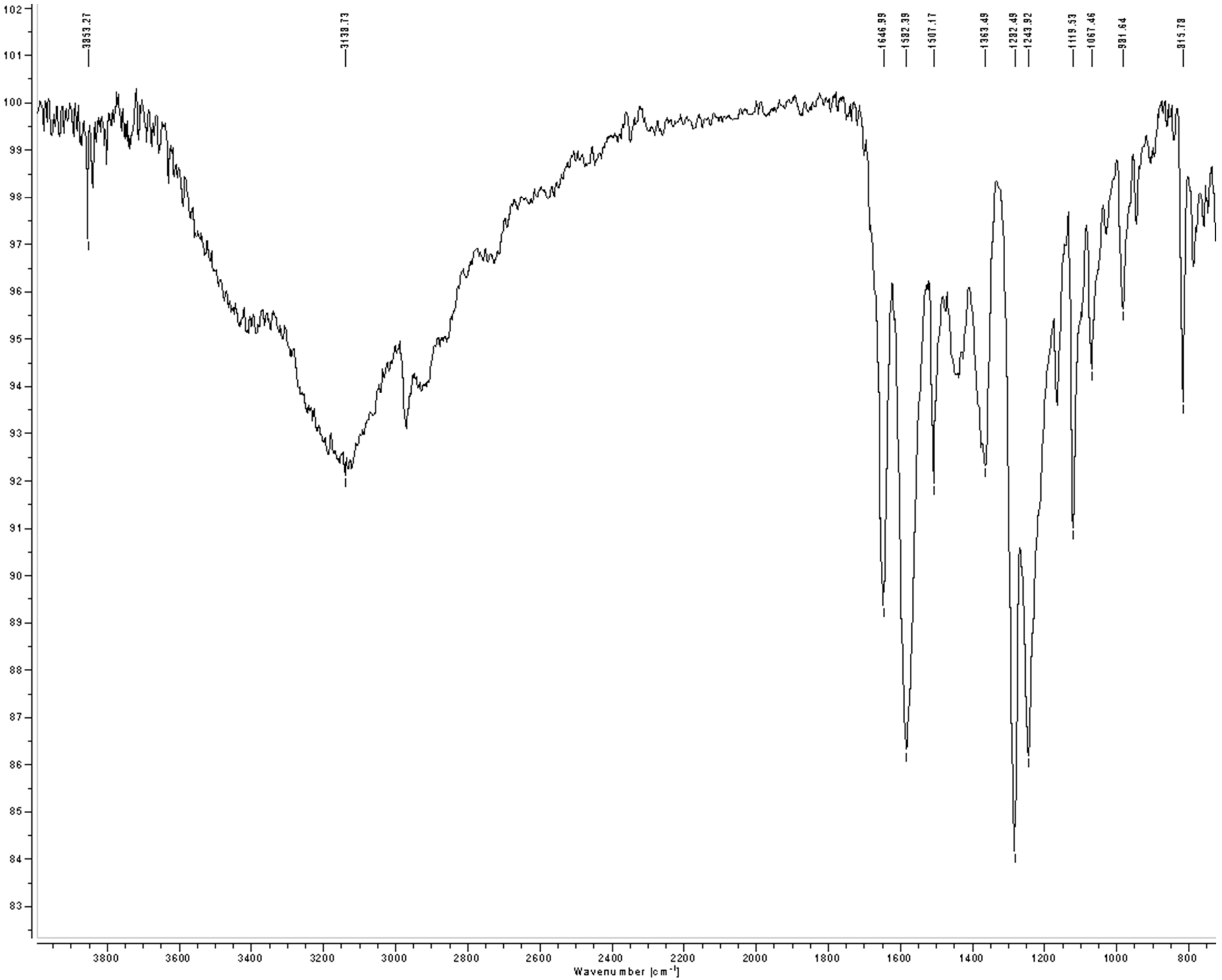

Figure S11. FT-IR Spectrum of 4-Hydroxy-3-(3-methyl-2-butenyl)acetophenone (KBr)

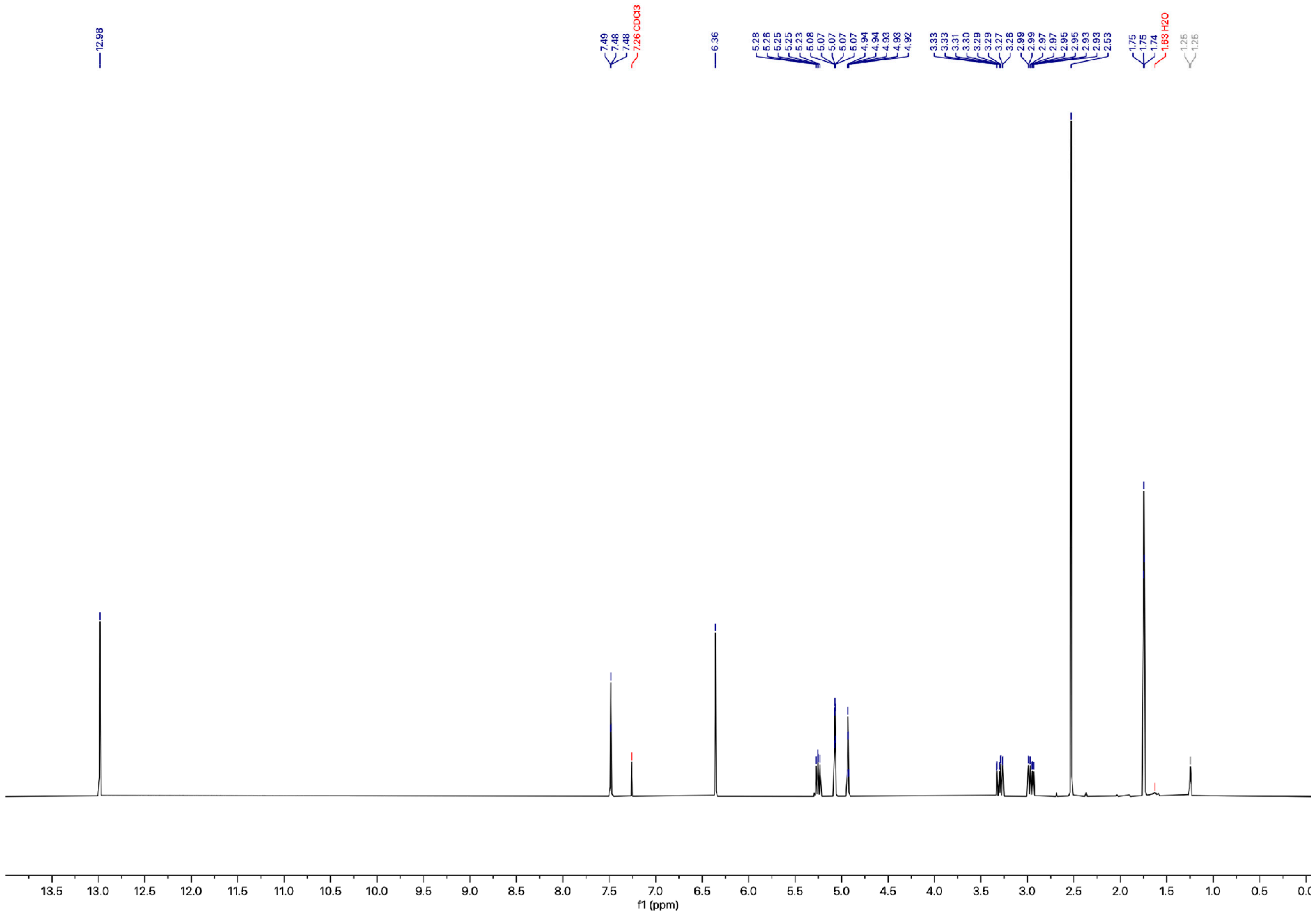

Figure S12. **^{1}H-NMR Spectrum of 6-Hydroxy-2-isopropenyl-5-acetyl-2,3-dihydrobenzofuran ($CDCl_3$, 400 MHz) δ 1.75 (t, *J* = 1.2 Hz, 3H), 2.53 (s, 3H), 2.96 (ddd, *J* = 15.4, 7.4, 1.4 Hz, 1H), 3.30 (ddd, *J* = 15.3, 9.5, 1.3 Hz, 1H), 4.93 (p, *J* = 1.5 Hz, 1H), 5.07 (dt, *J* = 1.7, 0.9 Hz, 1H), 5.21 – 5.32 (m, 1H), 6.36 (s, 1H), 7.48 (t, *J* = 1.4 Hz, 1H), 12.98 (s, 1H).**

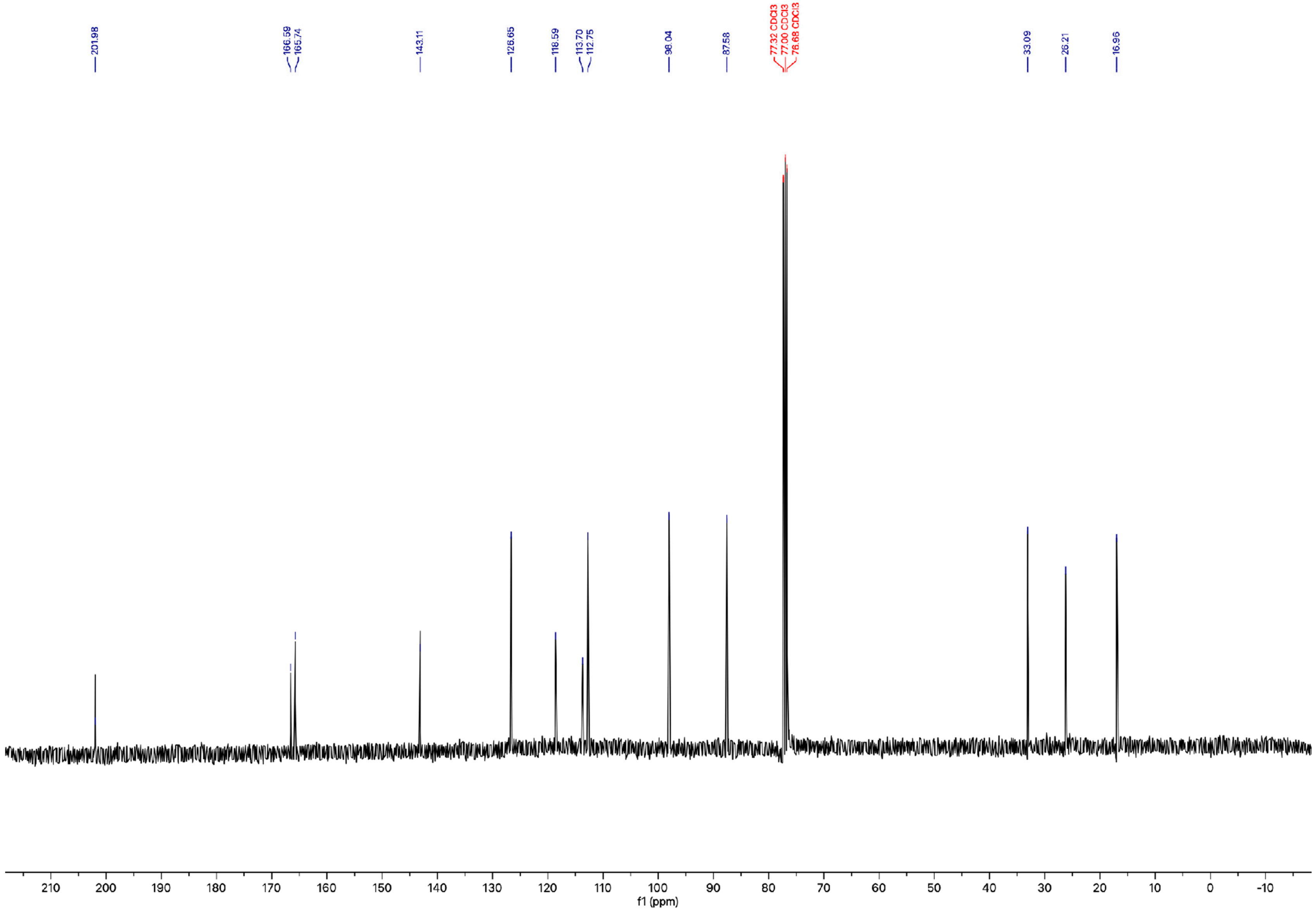

Figure S13. **^{13}C-NMR Spectrum of 6-Hydroxy-2-isopropenyl-5-acetyl-2,3-dihydrobenzofuran ($CDCl_3$, 101 MHz) δ 201.98, 166.59, 165.74, 143.11, 126.65, 118.59, 113.70, 112.75, 98.04, 87.58, 77.32, 77.00, 76.68, 33.09, 26.21, 16.96.**

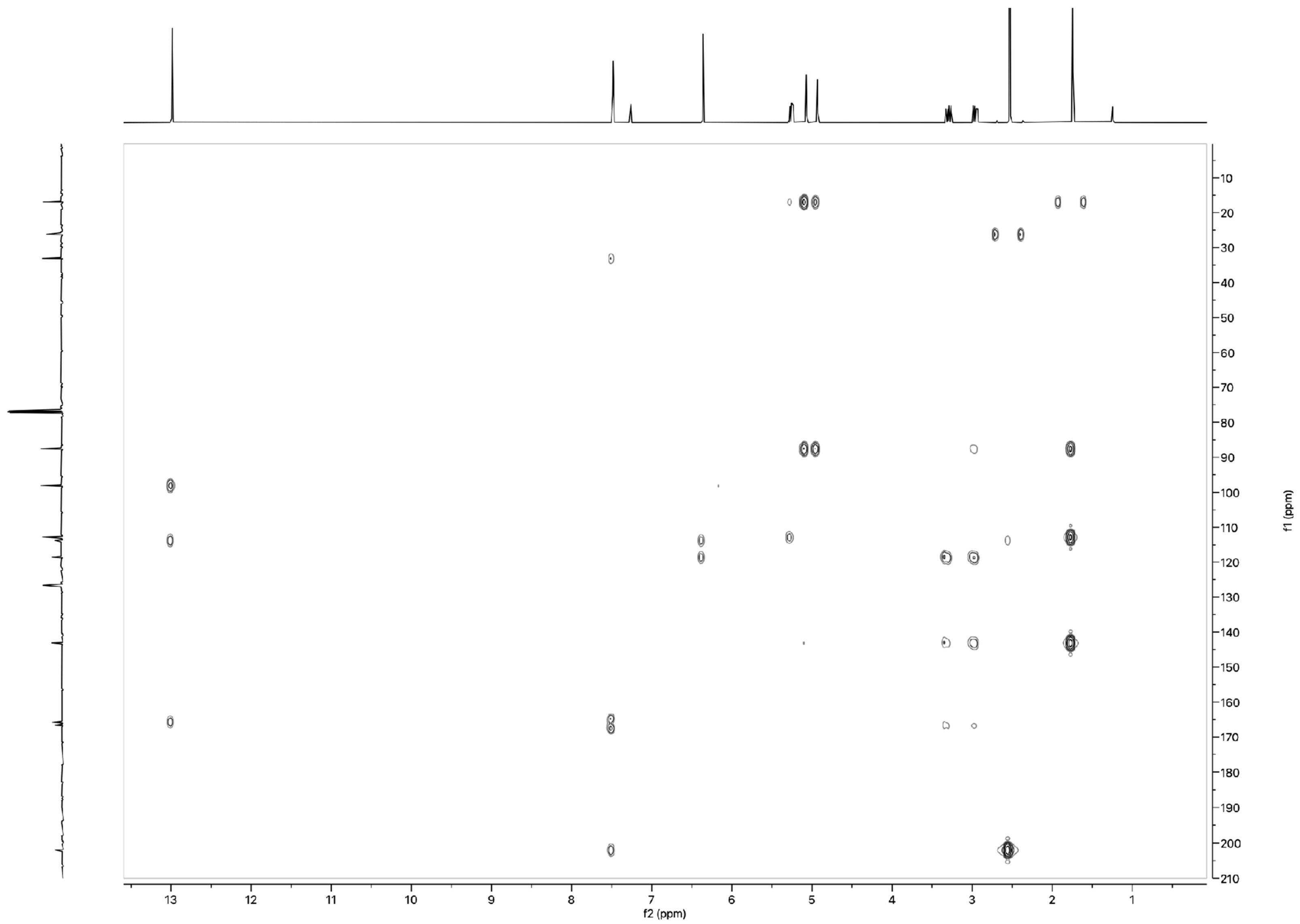

Figure S14. HSQC Spectrum of 6-Hydroxy-2-isopropenyl-5-acetyl-2,3-dihydrobenzofuran ($CDCl_3$)

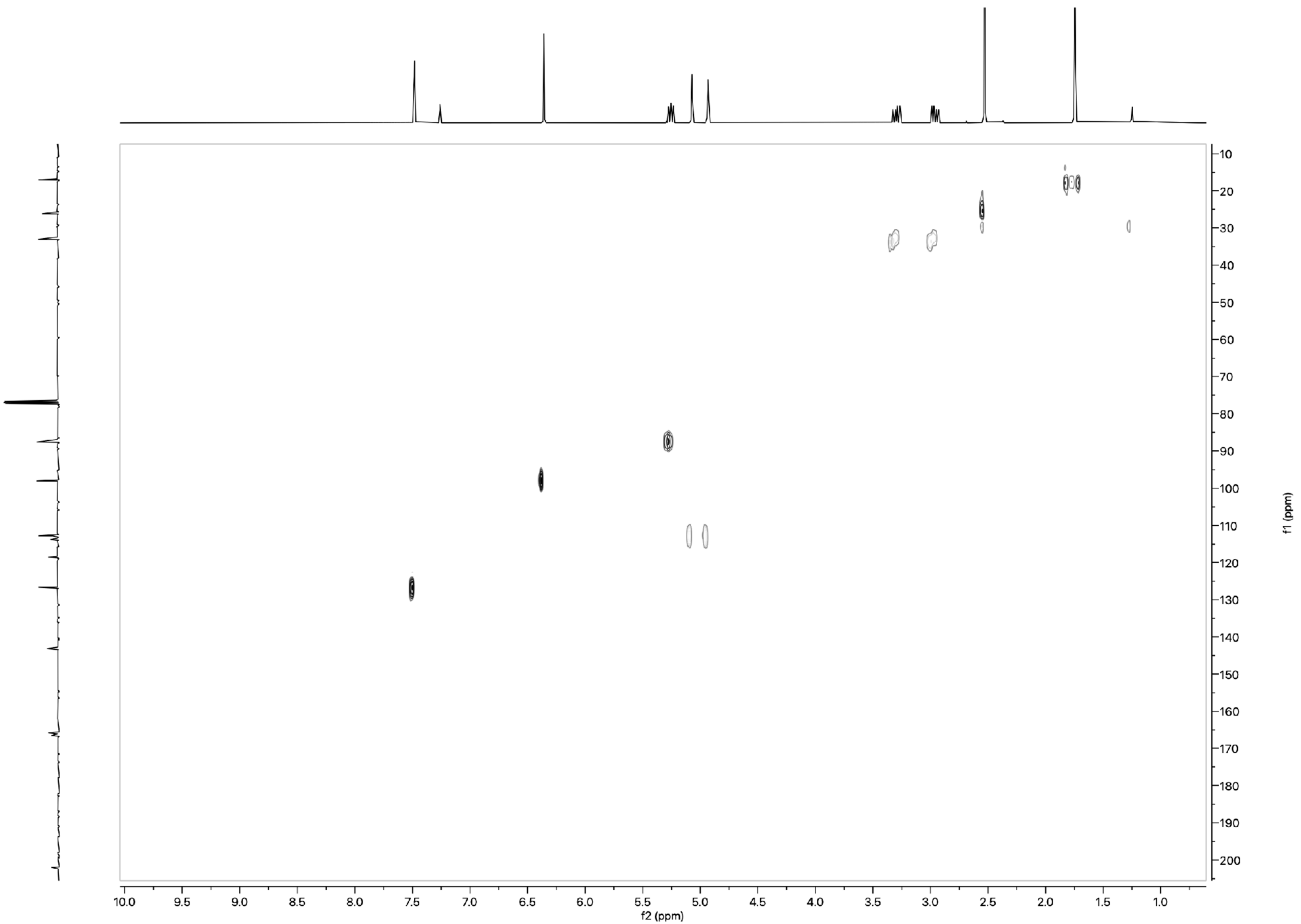

Figure S15. HMBC Spectrum of 6-Hydroxy-2-isopropenyl-5-acetyl-2,3-dihydrobenzofuran ($CDCl_3$)

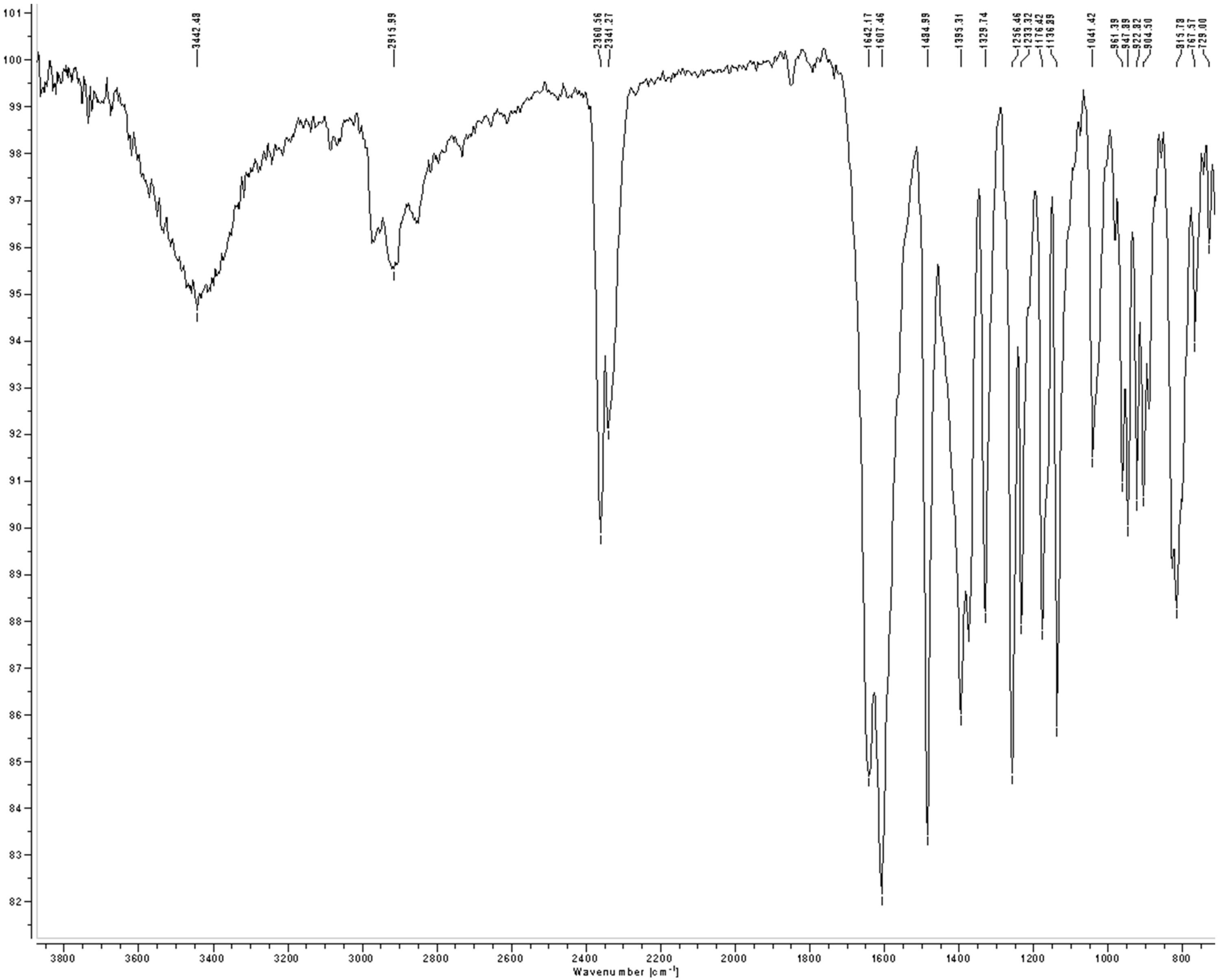

Figure S16. FT-IR Spectrum of 6-Hydroxy-2-isopropenyl-5-acetyl-2,3-dihydrobenzofuran (KBr)
